# Cellular and viral peptides bind multiple sites on the N‐terminal domain of clathrin

**DOI:** 10.1111/tra.12457

**Published:** 2016-12-14

**Authors:** Julia Muenzner, Linton M. Traub, Bernard T. Kelly, Stephen C. Graham

**Affiliations:** ^1^ Department of Pathology University of Cambridge Cambridge UK; ^2^ Department of Cell Biology University of Pittsburgh School of Medicine Pittsburgh PA; ^3^ Cambridge Institute for Medical Research, Department of Clinical Biochemistry University of Cambridge Cambridge UK

**Keywords:** amphiphysin, arrestin, assembly polypeptide 2 (AP2), clathrin‐mediated endocytosis, endocytosis, hepatitis D virus

## Abstract

Short peptide motifs in unstructured regions of clathrin‐adaptor proteins recruit clathrin to membranes to facilitate post‐Golgi membrane transport. Three consensus clathrin‐binding peptide sequences have been identified and structural studies show that each binds distinct sites on the clathrin heavy chain N‐terminal domain (NTD). A fourth binding site for adaptors on NTD has been functionally identified but not structurally characterised. We have solved high resolution structures of NTD bound to peptide motifs from the cellular clathrin adaptors β2 adaptin and amphiphysin plus a putative viral clathrin adaptor, hepatitis D virus large antigen (HDAg‐L). Surprisingly, with each peptide we observe simultaneous peptide binding at multiple sites on NTD and viral peptides binding to the same sites as cellular peptides. Peptides containing clathrin‐box motifs (CBMs) with the consensus sequence LΦxΦ[DE] bind at the ‘arrestin box’ on NTD, between β‐propeller blades 4 and 5, which had previously been thought to bind a distinct consensus sequence. Further, we structurally define the fourth peptide binding site on NTD, which we term the Royle box. In vitro binding assays show that clathrin is more readily captured by cellular CBMs than by HDAg‐L, and site‐directed mutagenesis confirms that multiple binding sites on NTD contribute to efficient capture by CBM peptides.

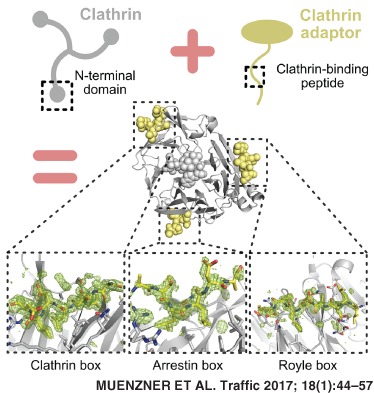

## Graphical abstract

Short peptide motifs in unstructured regions of clathrin‐adaptor proteins recruit clathrin to membranes to facilitate post‐Golgi membrane transport. A total of 3 consensus clathrin‐binding peptide sequences have been identified and structural studies show that each binds distinct sites on the clathrin heavy chain N‐terminal domain (NTD). A fourth binding site for adaptors on NTD has been functionally identified but not structurally characterized. We have solved high‐resolution structures of NTD bound to peptide motifs from the cellular clathrin adaptors β2 adaptin and amphiphysin plus a putative viral clathrin adaptor, hepatitis D virus large antigen (HDAg‐L). Surprisingly, with each peptide we observe simultaneous peptide binding at multiple sites on NTD and viral peptides binding to the same sites as cellular peptides. Peptides containing clathrin‐box motifs (CBMs) with consensus sequence LΦxΦ[DE] bind at the “arrestin box” on NTD, between β‐propeller blades 4 and 5, which had previously been thought to bind a distinct consensus sequence. Further, we structurally define the fourth peptide binding site on NTD, which we term the Royle box. in vitro binding assays show that clathrin is more readily captured by cellular CBMs than by HDAg‐L, and site‐directed mutagenesis confirms that multiple binding sites on NTD contribute to efficient capture by CBM peptides.

## 1 INTRODUCTION

Clathrin mediates vesicular transport between post‐Golgi membranes in eukaryotes and is targeted to specific membranes by interactions with clathrin adaptor proteins.^1^, ^2^, ^3^ Individually these interactions are weak,^4^ but because clathrin polymerization drives the growth of a network of available binding sites, a wide range of adaptors and accessory factors may be recruited and retained at sites of coated pit formation.^2^, ^5^, ^6^, ^7^

Clathrin:adaptor interactions are typically driven by linear peptide motifs in the unstructured regions of clathrin adaptors that bind the N‐terminal β‐propeller domain (NTD) of the clathrin heavy chain at several distinct sites (reviewed in reference 8): the “clathrin‐box motif” (CBM), consensus sequence LΦxΦ[DE] (where x denotes any amino acid, Φ denotes a bulky hydrophobic residue and [DE] is a glutamate or aspartate), binds in a groove between blades 1 and 2 of the NTD β‐propeller^3^, ^9^, ^10^; and the “W box” consensus PWxxW, binds the cleft near the centre of the NTD β‐propeller.^11^, ^12^ Thirdly, an extended surface loop of the arrestin 2 long isoform (arrestin2L) has been shown to occupy the “arrestin box”, a site lying between blades 4 and 5 of the NTD that binds peptides with consensus [LI][LI]GxL.^13^ More recently, a fourth adaptor binding site on the clathrin NTD, between blades 6 and 7, was defined by Willox and Royle^14^ on the basis of functional experiments in HeLa cells expressing clathrin heavy chain mutated in the NTD. This last study found that even a single functional NTD site was sufficient to sustain transferrin uptake.

The observation that any individual binding site on NTD is sufficient to sustain clathrin‐mediated endocytosis of the transferrin receptor raises several questions. Does it reflect promiscuity in the binding of clathrin‐interaction motifs, such that an individual clathrin‐binding motif can bind to different sites on NTD, or does it instead suggest intrinsic redundancy in the recruitment of adaptors such that endocytosis still proceeds even when an entire ‘class’ of clathrin‐binding motif is prevented from binding clathrin? Previous studies suggest the latter, as each peptide binding site on clathrin characterized to date has a distinct consensus binding motif.^10^, ^12^, ^13^ However, recent studies have suggested that the binding of peptides to clathrin may be promiscuous.^14^, ^15^ Promiscuity of CBM peptides for multiple sites on clathrin is relevant in the context of host:pathogen interactions, as it has previously been observed that viruses contain motifs resembling cellular CBMs that interact with clathrin in cells. These viral proteins have the ability to sequester clathrin, thus preventing endocytosis.^16^, ^17^ In the case of hepatitis D virus (HDV), which harbours a putative CBM sequence in the C‐terminal region of the large antigen protein (HDAg‐L), the presence of the clathrin binding motif seems essential for production of virus particles.^18^ If viral CBM peptides bind only to the clathrin box on NTD it would be possible to blockade this site using a small molecule inhibitor.^19^ This blockade would prevent virus hijacking of clathrin without perturbing cellular endocytosis, which can proceed when binding to the NTD “clathrin box” site is disrupted.^14^ However, if viral CBMs bind multiple sites on NTD with comparable affinities then small molecule interventions are unlikely to succeed. We thus sought to investigate the relative affinity of cellular vs viral peptides for clathrin NTD and to compare their modes of binding. Further, we sought to investigate the potential degeneracy of clathrin binding that had been suggested by previous studies.^14^, ^15^

Here we present high‐resolution structures of clathrin NTD bound to cellular and viral peptide motifs. Surprisingly, in all cases we observe peptide binding at multiple sites on NTD. We use clathrin‐binding assays and site‐directed mutagenesis to qualitatively assess the binding of these peptide motifs to the different sites on NTD. Further, we provide the first structural characterization of the putative “fourth” adaptor binding site on clathrin NTD.

## 2 RESULTS

### 2.1 Cellular clathrin‐binding motifs recruit clathrin more efficiently than those from hepatitis D virus

**Figure 1:**
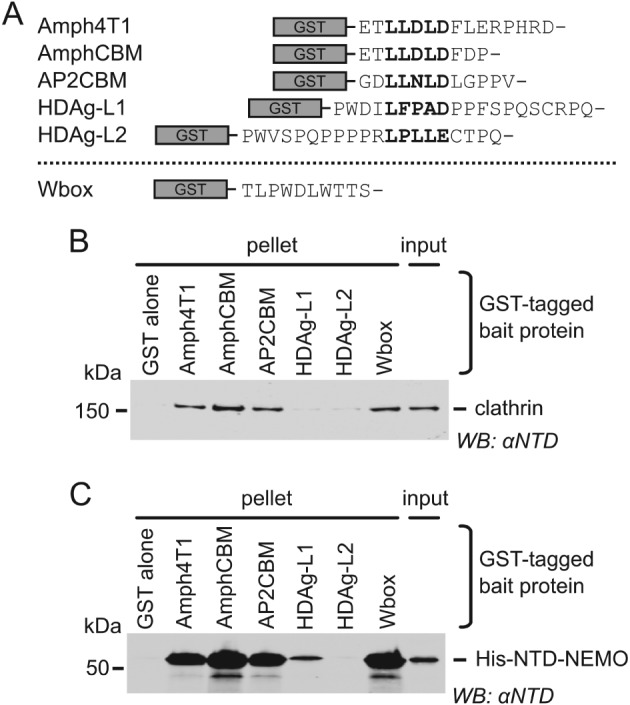
Cellular and viral peptide motifs bind clathrin N‐terminal domain (NTD) to different degrees. A, Glutathione S‐transferase (GST) fusions of clathrin‐binding peptides used in this study. Clathrin‐box motifs (CBMs) are aligned in bold. Amph4T1, human amphiphysin I CBM plus additional residues derived from the expression vector^22^; AmphCBM, human amphiphysin I CBM; AP2CBM, CBM from flexible hinge of β2 adaptin subunit of human AP2; HDAg‐L1, putative CBM from clade I hepatitis D virus large antigen; HDAg‐L2, putative CBM from clade II hepatitis D virus large antigen; Wbox, human amphiphysin W box binding motif. B, Capture (“GST pull‐down”) of purified clathrin by GST‐tagged clathrin‐binding peptides. Clathrin (input) was incubated with glutathione sepharose pre‐loaded with GST‐tagged “bait” proteins. After washing, proteins bound to the beads (pellet) were subjected to SDS‐PAGE and immunoblotting (WB) using an antibody that recognizes clathrin NTD (αNTD). C, Capture of His‐NTD‐NEMO by GST‐tagged clathrin binding peptides. Purified recombinant His‐NTD‐NEMO was used in GST pull‐down experiments as in (B).

The CBMs from the cellular proteins β2 adaptin (AP2CBM) and amphiphysin (AmphCBM),^9^, ^20^ the W box motif of amphiphysin (Wbox),^11^, ^12^, ^20^, ^21^ and the C‐terminal extensions of HDAg‐L from 2 different HDV genotypes containing putative clathrin binding motifs (HDAg‐L1 and HDAg‐L2, respectively)^16^, ^18^ were fused to glutathione S‐transferase (GST) (Figure 1A) and immobilized on glutathione resin for use in “GST pull‐down” experiments to capture clathrin purified from pig brain. In addition, to aid comparison with previous biochemical studies,^11^ an extended amphiphysin CBM construct (termed Amph4T1) was used in which the clathrin‐binding motif is followed by the amino acids “LERPHRD” arising from the XhoI cloning site and subsequent vector‐derived nucleotides.^22^ Consistent with previous studies,^11^, ^12^ clathrin was efficiently captured by GST fused to AP2CBM, AmphCBM, Amph4T1 or the amphiphysin Wbox, while it was not significantly captured by GST alone (Figure 1B). Interestingly, clathrin was not efficiently captured by GST fused to either of the HDAg‐L C‐terminal extensions tested (Figure 1B).

To facilitate subsequent mutational work the N‐terminal domain (NTD) of bovine clathrin heavy chain (100% amino acid identity to human clathrin heavy chain NTD), fused to a His_6_ affinity tag at the N terminus and to the dimerization domain of NF‐κB essential modulator (NEMO)^23^ at the C terminus, was purified following expression in *Escherichia coli*. This His‐NTD‐NEMO protein binds GST‐AP2CBM much more efficiently than His‐NTD lacking the NEMO oligomerization domain (Figure S1A). We ascribe this to increased avidity of binding, as His‐NTD‐NEMO is capable of oligomerizing at higher concentrations whereas His‐NTD is monomeric (Figure S1). As observed using purified clathrin, recombinant His‐NTD‐NEMO is efficiently pulled‐down by GST‐AP2CBM, GST‐AmphCBM, GST‐Amph4T1 and GST‐Wbox (Figure 1C). His‐NTD‐NEMO is weakly pulled down by GST‐HDAg‐L1, whereas GST‐HDAg‐L2 does not pull down His‐NTD‐NEMO any more efficiently than does GST alone.

### 2.2 CBMs of cellular and viral peptides bind multiple sites on the clathrin NTD

**Figure 2:**
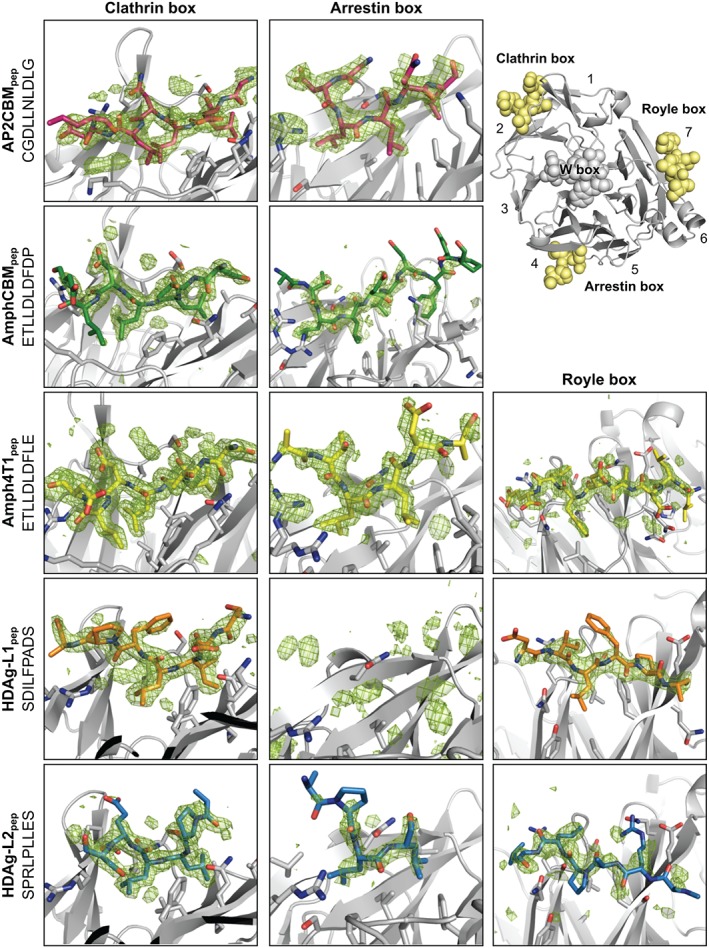
The clathrin‐box motifs (CBMs) of cellular and viral proteins bind multiple sites on clathrin N‐terminal domain (NTD). Unboxed image shows the β‐propeller fold of clathrin NTD (grey ribbons) with numbers enumerating the 7 β‐stranded blades. Spheres represent peptides bound at the 4 peptide‐interaction sites on NTD. Boxed images show CBM‐containing peptides (sticks, carbon atoms coloured as follows: AP2CBM_pep_, magenta; AmphCBM_pep_, dark green; Amph4T1_pep_, yellow; HDAg‐L1_pep_, orange; HDAg‐L2_pep_, light blue) bound at the clathrin box, arrestin box and Royle box sites on clathrin NTD (grey ribbons). Unbiased *F*_O_‐*F*_C_ electron density (3 σ) used to place peptides into the structures is shown as a green mesh. Selected side chain atoms of clathrin NTD are shown (sticks with grey carbon atoms).

To gain structural insight into the binding of cellular and viral peptides, recombinant NTD was crystallized in the presence of peptides corresponding to CBMs of β2 adaptin and amphiphysin, the CBM region of the non‐natural Amph4T1 sequence, and the putative CBMs of HDAg‐L1 and HDAg‐L2. These co‐crystallization experiments were performed using high concentrations (3.4‐3.6 mM) of clathrin‐binding peptide to ensure saturation of the peptide binding sites. The structures of all co‐crystals were solved and refined to high resolution (Table 1) and, surprisingly, in all cases electron density consistent with the presence of peptide bound to NTD could be observed at more than 1 locus on NTD (Figure 2).

**Table 1:** Crystallographic data collection and refinement. Values for the highest resolution shell are shown in parentheses

NTD:	AP2CBM_pep_	AmphCBM_pep_	Amph4T1_pep_	HDAg‐L1_pep_	HDAg‐L2_pep_
**Data collection**					
Space group	*C*222_1_	*C*2	*C*2	*C*2	*C*2
Cell dimensions					
*a*, *b*, *c* (Å)	108.1, 133.2, 77.9	140.0, 134.1, 78.0	137.8, 131.0, 79.1	136.2, 131.2, 77.9	136.9, 131.2, 78.5
α, β, γ (°)	90.0, 90.0, 90.0	90.0, 115.1, 90.0	90.0, 116.2, 90.0	90.0, 115.6, 90.0	90.0, 115.9, 90.0
Resolution (Å)	57.1–1.8 (1.81–1.76)	67.1–1.9 (1.93–1.88)	33.6–1.7 (1.74–1.70)	48.4–2.2 (2.21–2.15)	39.2–2.0 (2.00–1.96)
*R*_merge_	0.053 (1.538)	0.149 (1.092)	0.055 (0.751)	0.101 (0.930)	0.081 (0.581)
< I/σI>	15.8 (1.2)	6.5 (1.3)	13.1 (1.6)	9.6 (1.5)	7.2 (1.5)
CC_1/2_	1.000 (0.672)	0.993 (0.568)	0.999 (0.564)	0.996 (0.501)	0.996 (0.563)
Completeness (%)	100.0 (100.0)	100.0 (100.0)	98.2 (94.5)	99.7 (99.0)	94.6 (95.9)
Redundancy	7.5 (7.0)	5.1 (4.4)	3.9 (3.4)	4.5 (4.3)	2.5 (2.3)
**Refinement**					
Resolution (Å)	57.1–1.8 (1.81–1.76)	67.1–1.9 (1.93–1.88)	33.6–1.7 (1.74–1.70)	48.4–2.2 (2.21–2.15)	39.2–2.0 (2.01–1.96)
No. of reflections (work/free)	52,951/2740	99,868/5291	128,743/6564	63,339/3381	79,976/4224
*R*_work_/*R*_free_	0.176/0.205	0.204/0.234	0.158/0.185	0.175/0.207	0.169/0.193
Ramachandran favoured regions (%)	98.7	98.6	98.8	98.2	99.1
Ramachandran outliers (%)	0.0	0.0	0.0	0.0	0.0
No. of atoms					
Protein	2836	5845	5796	5634	5634
Glycerol	—	18	6	6	6
Peptide ligands	104	268	340	200	240
Water	403	803	1019	437	583
B‐factors					
Protein	36.1	22.0	26.2	39.8	32.0
Glycerol	—	36.5	21.8	32.7	20.9
Peptide ligands	52.4	45.1	48.7	70.2	61.0
Water	53.5	37.8	42.3	45.6	38.2
r.m.s. deviations					
Bond lengths (Å)	0.016	0.010	0.014	0.020	0.019
Bond angles (°)	1.719	1.396	1.619	1.897	1.917
PDB ID	5M5R	5M5S	5M5T	5M5U	5M5V

Abbreviations: NTD, N‐terminal domain; PDB, Protein data bank.

In all the structures presented a peptide could be observed at the “clathrin box”, lying between blades 1 and 2 of the NTD β‐propeller fold (“clathrin box”, Figure 2). For the cellular peptides (AP2CBM_pep_, AmphCBM_pep_ and Amph4T1_pep_) the binding is similar to that previously described,^10^ with the 3 leucine side chains of the CBM LΦxΦ[DE] consensus sequence occupying the hydrophobic pocket formed at the groove between the 2 blades. The viral peptides (HDAg‐L1_pep_ and HDAg‐L2_pep_) bound at a similar site on NTD. However, in both cases only 2 consecutive hydrophobic side chains could be observed in the hydrophobic pocket (“IL” and “LL” in the cases of HDAg‐L1_pep_ and HDAg‐L2_pep_, respectively). Interestingly, for both HDAg‐L1_pep_ and HDAg‐L2_pep_ the residues bound at the clathrin box do not match predictions based on alignments to the CBM consensus sequence (Figure 1)^16^, ^18^: in HDAg‐L1_pep_ residues “ILFPA” occupy the positions corresponding to the LΦxΦ[DE], whereas in HDAg‐L2_pep_ the residues “LLES”, including a C‐terminal serine residue that is non‐native and was added to the peptide to aid solubility, occupy the positions equivalent to the first 4 residues of the LΦxΦ[DE] consensus.

**Figure 3:**
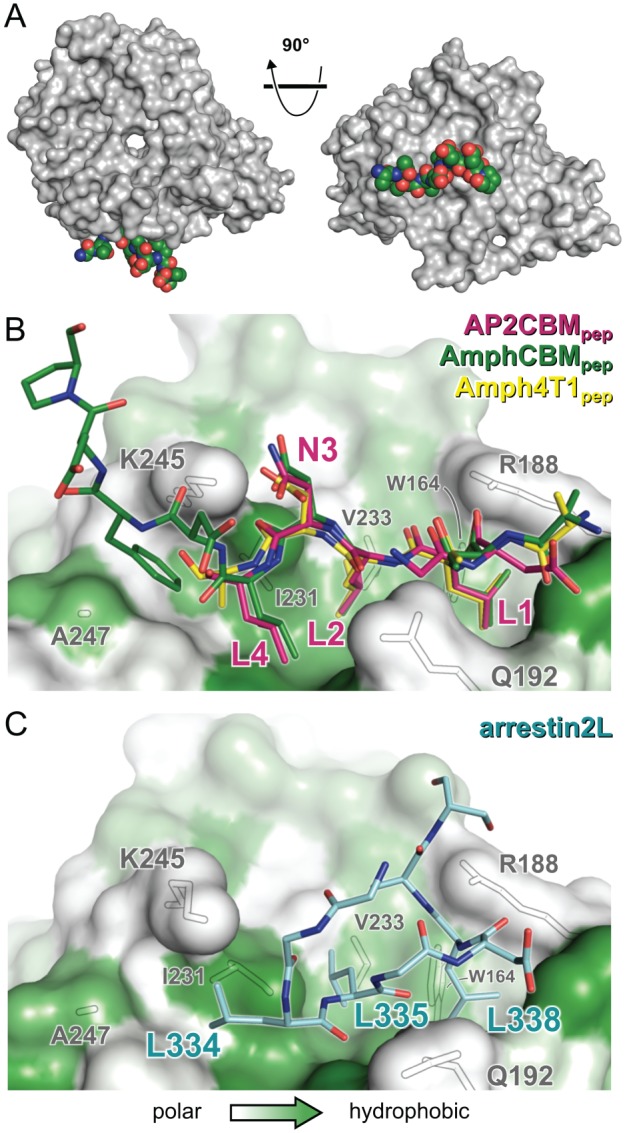
Cellular clathrin‐box motifs (CBMs) bind in a different conformation than arrestin2L at the arrestin box. A, The surface of clathrin N‐terminal domain (NTD) is shown (grey) oriented as in Figure 2 (left) and rotated by 90° around the horizontal axis (right). The AmphCBM_pep_ peptide bound at the arrestin box is shown as coloured spheres. B, Close‐up view of cellular CBM‐containing peptides bound at the arrestin box. Peptides are shown as sticks coloured as in Figure 2. The surface of clathrin NTD is shown, coloured from high (green) to low (white) surface residue hydrophobicity, with outlines of selected surface side chains shown in grey. Bound AP2CBM_pep_ residues are numbered by their position in the LΦxΦ[DE] CBM consensus sequence. C, The extended surface loop of arrestin 2 long isoform (arrestin2L) bound at the arrestin box (PDB 3GD1).^13^ NTD is shown as in (B) and arrestin2L residues 332‐340 are shown as sticks with cyan carbon atoms. Note that in (B) the direction of the bound peptides is right (N terminus) to left (C terminus), whereas in (C) the peptide chain between residues L334‐L338 runs in the opposite direction.

In addition to binding at the clathrin box, we observed significant binding of the cellular peptides (AP2CBM_pep_, AmphCBM_pep_ and Amph4T1_pep_) at the “arrestin box”, which lies between blades 4 and 5 of the NTD β‐propeller fold (“arrestin box”, Figure 2). While all 3 CBM peptides bind in the same general orientation at the arrestin box (Figure 3), the molecular details of these interactions differ from the interaction seen between NTD and the extended surface loop of the arrestin 2 long isoform (arrestin2L).^13^ Most notably, the directionality of the peptide chain is reversed. The first 2 leucine residues of each CBM (**LΦ**xΦ[DE]) bind in a hydrophobic cavity lined by the side chains of NTD residues W164, L183, S185, R188, V190, I194, F216, I231 and V233 plus the peptide backbones of Y184 and S191 (Figure 3B). The position occupied by these 2 peptide leucine side chains is very similar to that occupied by arrestin2L residues L338 and L335 (residues 5 and 2 of the [LI][LI]GxL arrestin box consensus motif, respectively) in the complex with NTD, despite the fact that in the arrestin2L:NTD complex the peptide backbone adopts a vastly different conformation to accommodate the 2 intervening amino acid residues (Figure 3C). In the structures presented here the side chain oxygen atom of NTD residue Q192 forms hydrogen bonds with the backbone amide protons of these 2 leucine residues (**LΦ**xΦ[DE]) and the side chain nitrogen atom of Q192 forms a hydrogen bond with the carbonyl oxygen of the second leucine (L**Φ**xΦ[DE]). While the electron density was not sufficiently well‐resolved to allow unambiguous modelling in the case of Amph4T1_pep_, for both the AP2CBM_pep_ and AmphCBM_pep_ structures the leucine side chain of the third CBM motif residue (LΦx**Φ**[DE]) occupies a similar location to the side chain of arrestin2L residue L334 ([**LI** ][LI]GxL), binding at a hydrophobic surface patch formed by NTD residues I194, F218, H229 and I231. In the AmphCBM_pep_ structure the side chain of the phenylalanine residue that follows the CBM forms an additional hydrophobic interaction with NTD, binding in a hydrophobic cleft formed by side chains of residues H229, I231 and A247, the aryl side chain region of K245, and the peptide backbones of residues 245‐247. The HDAg‐L2_pep_ peptide could also be observed binding at the arrestin site, although the interaction was less extensive (Figure 2). The binding was largely similar to that observed for the cellular CBM peptides: the side chains of the 2 consecutive leucine residues bound at the hydrophobic cleft and their backbone atoms interacted with the side chain of Q192 as described above. There was not strong evidence for the HDAg‐L1_pep_ peptide bound at the arrestin box (Figure 2).

### 2.3 Structural identification of the “fourth” peptide binding site on the clathrin NTD

**Figure 4:**
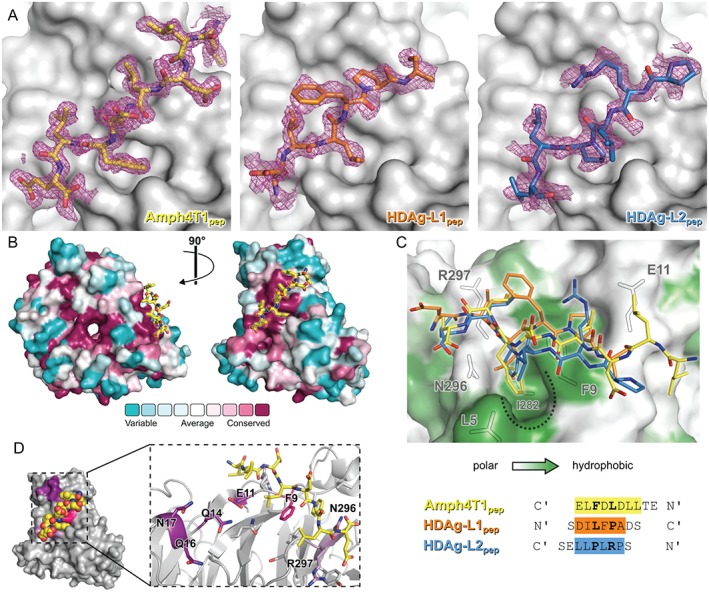
Localization and characterization of the fourth peptide binding site on clathrin N‐terminal domain (NTD): The Royle box. A, Amph4T1_pep_ (left), HDAg‐L1_pep_ (middle) and HDAg‐L2_pep_ (right) peptides bound at the Royle box in feature‐enhanced maps^24^ calculated using the final refined model (2σ, magenta). For clarity, maps are shown only within 2 Å of the bound peptides. Peptides are shown as sticks, coloured as in Figure 2, and clathrin NTD is shown as a grey surface. B, The surface of clathrin NTD, coloured by amino acid conservation from conserved (magenta) to variable (blue), is shown oriented as in Figure 2 (left) and rotated by 90° around the vertical axis (right). The Amph4T1_pep_ peptide bound at a conserved surface patch between NTD β‐propeller blades 6 and 7 (which we term the Royle box) is shown as sticks with yellow carbon atoms. C, Close‐up view of cellular and virus peptides bound at the Royle box. Peptides are shown as sticks coloured as in Figure 2. The surface of clathrin NTD is shown, coloured from high (green) to low (white) surface residue hydrophobicity, with outlines of selected surface side chains shown in grey. A hydrophobic NTD surface pocket that is occupied by hydrophobic residues of all three peptides is marked by a dotted line. The peptide sequences used for co‐crystallization are structurally aligned at the bottom of the panel. The directionality of the bound peptides is indicated. Residues that could be confidently modelled in the structures are highlighted and residues that form side chain interactions with NTD surface pockets are printed in bold type. D, Cellular and viral peptides bind near NTD residues functionally implicated in clathrin‐mediated endocytosis. The surface of NTD (grey) is oriented as in the right image of (A) with residues mutated by Willox and Royle^14^ (light and dark purple) or in this study (pink) highlighted. The Amph4T1_pep_ peptide is shown as spheres. Inset shows the Amph4T1_pep_ peptide (sticks with yellow carbon atoms) bound to NTD (grey, ribbon with selected side chains shown as sticks). The carbon atoms of residues substituted in clathrin mutants that disrupt transferrin uptake,^14^ a proxy for clathrin‐mediated endocytosis, are dark purple while those of residues where substitution does not affect transferrin uptake are light purple. The side chain of F9, mutated in this study, is coloured bright pink.

In addition to binding at the clathrin and arrestin boxes, in 3 of the NTD:peptide co‐crystal structures solved (Amph4T1_pep_, HDAg‐L1_pep_ and HDAg‐L2_pep_) a bound peptide could be observed lying across the interface between blades 6 and 7 of the NTD β‐propeller (Figure 2). This peptide binding site overlaps with the region identified in the functional studies of Willox and Royle^14^ as the fourth and final clathrin adaptor binding site on NTD, and we thus henceforth refer to it as the “Royle box” In comparison to surrounding residues, peptides were generally less well‐ordered when bound at this site than when bound at the clathrin box (Table S1). However, in all cases a single orientation of the peptide could be modelled with good stereochemistry and an acceptable fit to electron density (Figure 4A).

The surface residues bound by peptides at the Royle box are highly conserved amongst eukaryotic clathrin sequences (Figure 4B). Binding at the Royle box (Figure 4C) centres on a deep hydrophobic pocket lying at the interface of blades 6 and 7, formed by the side chains of NTD residues L5, I7, F9, I282, N296 and V327. In the co‐structure with Amph4T1_pep_ a phenylalanine side chain projects deep into this pocket while in the HDAg‐L1_pep_ and HDAg‐L2_pep_ structures the side chains of a leucine residue and proline residue, respectively, bind less deeply. In all structures a hydrophobic side chain (leucine in Amph4T1_pep_ and HDAg‐L2_pep_, isoleucine in HDAg‐L1_pep_) covers a surface hydrophobic patch formed by the hydrophobic portion of the R297 side chain and the hydrophobic faces of the peptide bonds between NTD residues 298‐300 on the surface of β‐propeller blade 6, and in all structures the backbone carbonyl oxygen of R297 forms a hydrogen bond with a backbone amide nitrogen of the bound peptide. In each structure 3 consecutive amino acid residues wrap around the side chain of F9 on the surface of blade 7, forming hydrophobic interactions with both faces of the phenylalanine residue's hydrophobic side chain benzyl group. In addition, in the co‐structures with HDAg‐L1_pep_ and Amph4T1_pep_ the backbone carbonyl oxygen of F9 forms a hydrogen bond with a backbone amide nitrogen of the bound peptide, and backbone atom(s) of E11 form hydrogen bond(s) with the bound peptide (1 bond in the case of HDAg‐L1_pep_, 2 in the case of Amph4T1_pep_). Despite the similar molecular interactions made between the Royle box and the bound peptides, we note that the direction of the peptide chain differs between HDAg‐L1_pep_ and Amph4T1_pep_/HDAg‐L2_pep_ (Figure 4C). In addition, unlike the binding of peptides to the clathrin and arrestin boxes, where the same side chains of the CBM sequence form key interactions, we note that residues outside the CBM consensus sequence also form extensive interactions at the Royle box.

To investigate whether the absence of AmphCBM_pep_ binding at the Royle box arose from an absence of stabilizing residues C‐terminal to the CBM motif, we solved the structure of NTD in complex with a longer sequence containing the human amphiphysin I CBM (AmphCBMlong_pep_, sequence ETLLDLDFDPFK; Table S2). As observed for AmphCBM_pep_, AmphCBMlong_pep_ bound NTD at the CBM and arrestin boxes but not at the Royle box (Figure S2).

### 2.4 Multiple interaction sites on clathrin NTD contribute to peptide binding in vitro

**Figure 5:**
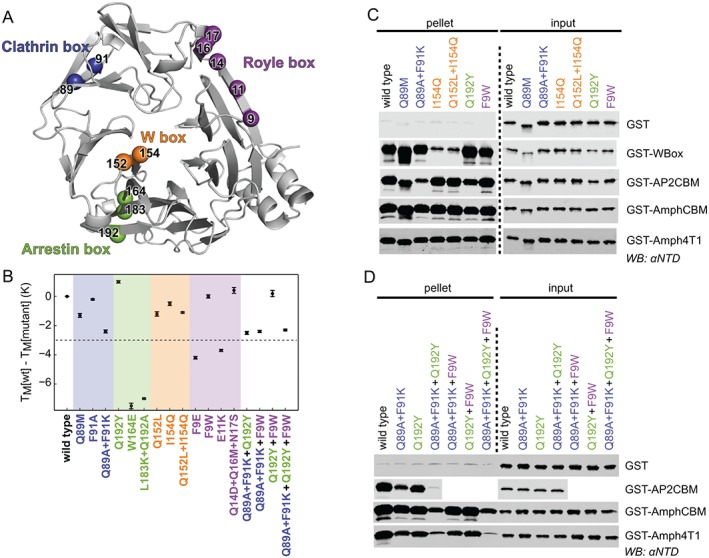
Mutation at single or multiple sites on clathrin N‐terminal domain (NTD) disrupts binding to peptide motifs. A, Ribbon representation of NTD (grey) showing the location of residues that were mutated on their own or in combination to disrupt peptide binding at the clathrin box (blue), arrestin box (green), W box (orange) and Royle box (purple). B, Thermal stability of single‐ or multiple‐site mutations of NTD as determined by differential scanning fluorimetry. The melting temperatures (T_M_) of mutants relative to that of wild‐type His‐NTD are shown (error bars represent the standard deviation of measurement in triplicate). Mutations that perturb the T_M_ by more than 3 K (dotted line) were not considered further. C and D, Capture of NTD mutants by glutathione S‐transferase (GST)‐tagged clathrin‐binding peptides. Purified recombinant wild‐type or mutant His‐NTD‐NEMO was incubated with glutathione sepharose pre‐loaded with GST‐tagged “bait” proteins. After washing, proteins bound to the beads (pellet) were subjected to SDS‐PAGE and immunoblotting (WB) using an antibody that recognizes clathrin NTD (αNTD).

A series of His‐NTD‐NEMO constructs with mutations at each of the 4 peptide binding sites on NTD were generated to probe whether all the interactions observed in the crystal structures contribute to binding in a biochemical context, or whether binding can be explained by a single dominant binding interaction. The mutations introduced at each site were informed by the crystal structures presented above and by previous studies (Figure 5A and Table 2). To confirm that these mutations did not introduce defects in NTD folding, the secondary structure and thermal stability of these mutants was probed by circular dichroism (CD) spectroscopy and differential scanning fluorimetry (DSF, a.k.a. Thermofluor), respectively. The CD spectra of mutants were very similar to that of wild‐type NTD (Figure S3), confirming that they had the correct secondary structure composition. However, DSF showed a number of mutants to have melting temperatures significantly lower than the wild‐type protein, consistent with disrupted folding, and these mutants were thus not considered further (Figure 5B).

**Table 2:** Clathrin heavy chain N‐terminal domain mutations

Mutation	Site	Reference
Q89M	Clathrin box	25
F91A	Clathrin box	25
Q89A + F91K	Clathrin box	This study
Q192Y	Arrestin box	This study
W164E	Arrestin box	13
L183K + Q192A	Arrestin box	This study
Q152L	W box	12
I154Q	W box	12
Q152L + I154Q	W box	14
F9E	Royle box	This study
F9W	Royle box	This study
E11K	Royle box	14
Q14D + Q16M + N17S	Royle box	14

A selection of the correctly folded mutants was tested for ability to bind the cellular clathrin‐binding motifs in GST pull‐down experiments. Given the modest ability of GST‐HDAg‐L1 and ‐L2 to capture His‐NTD‐NEMO (Figure 1C) we limited our analysis to the cellular peptide sequences (GST‐AP2CBM, GST‐AmphCBM, GST‐Amph4T1 and GST‐Wbox). Figure 5C shows that mutations at the W box (I154Q and I152L + I154Q) severely disrupt the ability of GST‐Wbox to capture His‐NTD‐NEMO, consistent with previous studies.^12^ His‐NTD‐NEMO with mutations at the clathrin box was less efficiently captured by GST‐AP2CBM, the defect being most pronounced for the Q89A + F91K mutant, but capture of these mutants by GST‐AmphCBM or GST‐Amph4T1 was largely unperturbed (Figure 5C). Similarly, His‐NTD‐NEMO mutated at the arrestin box (Q192Y) was captured less efficiently by GST‐AP2CBM but the capture of this mutant by GST‐AmphCBM or GST‐Amph4T1 was not significantly changed (Figure 5C).

To test whether mutation at more than 1 peptide binding site further reduced capture by cellular CBM peptides, His‐NTD‐NEMO constructs with mutations at multiple binding loci were generated. All these ‘compound mutants’ had CD spectra similar to that of the wild‐type protein (Figure S3) and melting temperatures within 3 K of the wild‐type protein (Figure 5B), consistent with the compound mutants being well‐folded. As shown in Figure 5D, while His‐NTD‐NEMO mutated at either the clathrin box (Q89A + F91K) or arrestin box (Q192Y) can still be captured by GST‐AP2CBM, combining the mutations (Q89A + F91K + Q192Y) reduces the binding to the level of the GST control. Similarly, His‐NTD‐NEMO mutated at both the clathrin and arrestin boxes is captured less efficiently by GST‐Amph4T1 than is the wild‐type protein or protein with mutants at either site individually. The decrease in capture of His‐NTD‐NEMO with mutated clathrin and arrestin boxes by GST‐Amph4T1 is more pronounced than is the capture of clathrin and Royle box or arrestin plus Royle box mutants. However, when NTD is mutated at all 3 sites, namely the arrestin, clathrin and Royle boxes, the extent of capture by GST‐Amph4T1 is further decreased and approaches the levels seen for GST alone. GST‐AmphCBM captures His‐NTD‐NEMO mutated at both the clathrin and arrestin boxes less efficiently than it does wild‐type protein or His‐NTD‐NEMO with either site mutated individually. However, none of the His‐NTD‐NEMO mutations tested completely abolished binding to GST‐AmphCBM.

### 2.5 The arrestin motif of AP2 can also bind multiple sites on clathrin NTD

**Figure 6:**
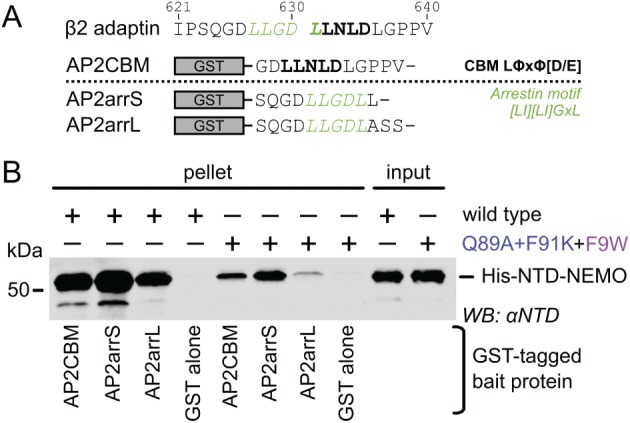
The overlapping β2 adaptin arrestin‐box and clathrin‐box motifs both bind multiple sites on clathrin N‐terminal domain (NTD). A, Glutathione S‐transferase (GST) fusions of the clathrin‐box motif (GST‐AP2CBM) and arrestin‐binding motif (GST‐AP2arrS and GST‐AP2arrL) from the hinge region of β2 adaptin, the arrestin‐box motif constructs having either the next residue of β2 adaptin (“L”, GST‐AP2arrS) or the sequence that follows the LLGDL motif of arrestin2L (“ASS”, GST‐AP2arrL) appended at their C termini. B, Capture of wild‐type NTD or a mutant with disrupted clathrin and Royle boxes (Q89A + F91K + F9W) by GST‐tagged β2 adaptin clathrin‐binding motifs. Purified recombinant wild‐type or mutant His‐NTD‐NEMO was incubated with glutathione sepharose pre‐loaded with GST‐tagged “bait” proteins. After washing, proteins bound to the beads (pellet) were subjected to SDS‐PAGE and immunoblotting (WB) using an antibody that recognizes clathrin NTD (αNTD).

The hinge region of the assembly polypeptide 2 (AP2) complex β2 adaptin subunit contains 2 overlapping clathrin‐binding motifs, a CBM and an arrestin‐box motif (Figure 6A). However, the CBM motif can bind at the arrestin box in addition to binding at the clathrin box (Figure 2) and both such interactions contribute to NTD recruitment (Figure 5D). We therefore sought to compare the NTD binding of the β2 adaptin arrestin‐box motif to that of the β2 adaptin CBM.

Two GST‐tagged peptide constructs containing the arrestin‐box motif of β2 adaptin (GST‐AP2arrL and GST‐AP2arrS) were generated (Figure 6A). Both contained the β2 adaptin arrestin‐box motif (LLGDL) but, to avoid the potentially confounding issue of a carboxylate group immediately following the final residue of the motif, the sequences of their C‐terminal residues differed: AP2arrS had the subsequent “L” residue of β2 adaptin appended to the arrestin‐box motif, while AP2arrL had the residues “ASS” appended, corresponding to the residues that follow the LLGDL arrestin‐box motif of arrestin2L.^13^ We compared the ability of GST‐AP2CBM, GST‐AP2arrS and GST‐AP2arrL to capture either wild‐type His‐NTD‐NEMO or a mutant (Q98A + F91K + F9W) where the clathrin and Royle boxes, but not the arrestin box, had been disrupted. Figure 6B shows that GST‐AP2CBM and GST‐AP2arrS capture wild‐type and Q98A + F91K +F9W His‐NTD‐NEMO more efficiently than does AP2arrL, suggesting that the arrestin‐box motif (LLGDL) alone binds the arrestin box more weakly than does the CBM or an extended arrestin motif (LLGDLL) containing the first 2 residues of the overlapping CBM. However, these experiments also show that GST‐AP2arrL and GST‐AP2arrS capture wild‐type His‐NTD‐NEMO much more efficiently than they do the Q98A + F91K + F9W mutant, consistent with the β2 adaptin arrestin‐box motif binding to either the clathrin or Royle boxes in addition to the arrestin box.

## 3 DISCUSSION

This study presents the structure of the clathrin heavy chain NTD solved in the presence of cellular and viral clathrin‐binding peptides. In all cases, we observe that these peptides bind promiscuously to more than one site on the clathrin NTD surface. This differs from previous high‐resolution structural characterizations of peptide binding to clathrin NTD: structures of NTD solved in the presence of β3 adaptin and β‐arrestin 2 CBMs showed binding only at the clathrin box,^10^ only the W box site is occupied in the structure solved in the presence of a peptide derived from the “second” (PWDLW) clathrin‐binding motif of amphiphysin,^12^ and the structure of NTD in complex with a long splice form arrestin 2 (arrestin2L) shows binding of 2 different peptide motifs at the clathrin and arrestin box sites. We observe that the CBMs of β2 adaptin, amphiphysin and HDAg‐L2 bind to both the clathrin and arrestin box sites. Further, we provide the first structural characterization of the putative fourth and final peptide binding site on clathrin NTD,^14^ which we term the Royle box.

### 3.1 The arrestin box binds linear CBM peptides

The structure of arrestin2L bound to clathrin NTD revealed two different peptide epitopes bound at the clathrin and arrestin boxes.^13^ The epitope bound at the arrestin box comprised an 8 amino acid surface loop that connects 2 adjacent anti‐parallel beta strands of arrestin2L: this loop thus necessarily forms a relatively tight turn at its apex. The key molecular interactions are formed by three leucine side chains (L334, L335 and L338) from the arrestin2L loop that are adjacent in space (Figure 3C), but not consecutive in sequence, and biochemical studies defined the consensus binding sequence of this loop as [LI][LI]GxL.^13^

The structures presented here show that CBM peptides, matching the LΦxΦ[DE] CBM consensus sequence, also bind NTD at the arrestin box (Figure 2) and that this interaction contributes to binding in vitro (Figure 5). Interestingly, the molecular determinants of binding are conserved between the arrestin2L loop and the CBM peptides: hydrophobic leucine or isoleucine side chains bind the groove between NTD β‐propeller blades 4 and 5, occupying roughly equivalent spatial positions (Figure 3). However, the peptide backbone topology differs substantially as does the spacing between the crucial leucine/isoleucine residues. We therefore propose that the consensus sequence for binding at the arrestin box is likely to be context‐dependent. In the case of arrestin2L, the amino acids that mediate binding are partly determined by the constrained nature of the surface loop. However, when presented as linear motifs, as is likely to be the biological context of the β2 adaptin, epsin 1 and amphiphysin CBM epitopes,^3^, ^4^, ^9^, ^21^, ^26^ peptides that conform to the LΦxΦ[DE] CBM consensus sequence can also bind the arrestin box.

Our structural characterization of CBM peptides bound promiscuously at multiple sites on clathrin NTD is largely consistent with a recent biophysical study showing promiscuous binding of long peptides derived from β2 adaptin and AP180 at the clathrin, arrestin and W boxes of NTD.^15^ However, while the long β2 adaptin peptide used in the biophysical study harboured both CBM (LLNLD) and arrestin2L (LLGDL) consensus sequences, the β2 adaptin CBM (AP2CBM) peptide used here contains only a CBM. We observe that combining mutations at the arrestin and clathrin boxes completely abolishes the ability of the AP2CBM to capture NTD in GST pull‐down assays, confirming that CBM peptides bind promiscuously to both sites. Unlike the previous biophysical study, peptide binding at the W box was not observed in any of the crystal structures presented here. We ascribe this to the use of much longer peptides in the biophysical study that may harbour additional, non‐canonical W box binding motifs.

Given the similar molecular determinants of linear peptide binding at the clathrin and arrestin boxes, it is perhaps surprising that binding at the arrestin box was not observed in the previous co‐crystallization study that used the β3 adaptin and β‐arrestin 2 CBM peptides.^10^ However, we note the prior study used a lower molar excess of peptide (4‐fold excess versus 7‐ to 10‐fold excess used here). Further, the extended cryo‐protection protocols employed in the prior study, using cryo‐preservative solutions without added peptide, may have facilitated dissociation of bound peptides from lower‐affinity sites and thus removed evidence of their binding.

### 3.2 Structural characterization of the Royle box

As observed at the arrestin box (above), the molecular determinants of binding at the Royle box are conserved despite a difference in peptide orientation observed for the bound HDAg‐L1_pep_ peptide vs the bound HDAg‐L2_pep_ and Amph4T1_pep_ peptides. However, unlike at the arrestin box, residues that form the molecular interactions at the Royle box do not correspond to those conserved in the LΦxΦ[DE] consensus CBM sequence and we note that several peptides containing a CBM sequence (AP2CBM_pep_, AmphCBM_pep_ and AmphCBMlong_pep_) do not bind at the Royle box (Figures 2 and S2). Together, this suggests that a distinct consensus sequence mediates binding of cellular proteins at the Royle box. While the HDAg‐L1_pep_ and HDAg‐L2_pep_ peptides that bind the site occur naturally in hepatitis D virus, the Amph4T1_pep_ sequence used in this study contains amino acids corresponding to those introduced when cloning the amphiphysin CBM into the pGEX‐4T1 vector.^22^ Attempts to define a consensus sequence and screen in silico for genuine cellular binding peptide motifs were unsuccessful due to the degeneracy of the peptide sequences bound in our structures. Identification of the Royle box consensus binding motif therefore awaits experimental elucidation.

In accordance with previous functional studies,^14^ all three structures of peptides bound at the Royle box presented here show binding at a conserved surface patch that lies between blades 6 and 7 of the NTD β‐propeller (Figure 4B). The bound peptides all wrap around the hydrophobic side chain of NTD residue F9, which also lines the hydrophobic pocket central to the interaction of peptides at the Royle box (Figures 4C and 4D). Mutation of F9 to the bulkier residue tryptophan does not destabilize NTD (Figure 5B) but is able to diminish binding to GST‐Amph4T1 when combined with mutations at the arrestin and clathrin boxes, confirming the importance of F9 for peptide binding. Previous functional experiments showed that 2 sets of mutations at the Royle box, E11K and Q14D + Q16M + N17S (Figure 4D), were sufficient to prevent transferrin uptake (a readout for clathrin‐mediated endocytosis) when combined with mutations at the clathrin, arrestin and W boxes. While the side chain of E11 does not interact directly with bound peptides, in the HDAg‐L1_pep_ and Amph4T1_pep_ structures E11 backbone atoms form hydrogen bonds with the bound peptide. Further, we find that the E11K mutation reduces the T_M_ of NTD by 3.7 K, consistent with some destabilization of the protein fold. We propose that destabilization of the local fold in the E11K NTD mutant prevents its binding to Royle box binding epitopes in cells. Residues of the second disrupting mutation, Q14D + Q16M + N17S, do not directly contact peptides bound at the Royle box in our structures but are in close proximity to peptide‐binding residues (Figure 4D). Residue Q14 lies on the same short stretch of β‐sheet as E11, the side chains of these two residues forming a hydrogen bond, while Q16 and N17 lie on the surface of a short α‐helix immediately following Q14 (Figure 4D). The thermal stability of purified Q14D + Q16M + N17S NTD is slightly higher than the wild‐type NTD (Figure 5B), which may indicate perturbation of the protein fold in the vicinity of bound peptide. Alternatively, one could speculate that the N‐terminal residues of a bona fide cellular Royle box binding motif could bind the Royle box in an extended conformation and interact with these residues, although identification of such a motif remains elusive. A third set of mutations at the Royle box, N296A + R297E, did not seem to affect the ability of NTD to promote transferrin uptake,^14^ yet both residues are in close proximity to the peptide bound at the Royle box in the structures presented here. It is possible that the precise nature of substitutions introduced at residues N296 and R297 led to sustained transferrin uptake: N296 lines the rim of the deep hydrophobic pocket and its mutation to alanine, conferring a short hydrophobic side chain, should not prevent binding. Similarly, interactions with R297 are mediated primarily by the backbone and hydrophobic C^β^ and C^γ^ side chain atoms, all of which would remain when the residue was mutated to glutamate.

### 3.3 HDV peptides bind the same sites on NTD as cellular peptides

Previous studies showed that GST fused to residues 198‐210 of HDAg‐L, comprising the majority of the HDAg‐L C‐terminal extension that is expressed following editing of the HDV RNA antigenome,^27^ is capable of capturing clathrin heavy chain from cell lysates.^16^ Further, mutation of a putative CBM sequence in this C‐terminal extension prevented both co‐immunoprecipitation of clathrin heavy chain from transfected cells and the formation of virus‐like particles (VLP).^18^ It was therefore concluded that HDAg‐L is a novel viral clathrin adaptor‐like protein.^16^, ^18^ We sought to extend this observation by probing whether cellular and HDAg‐L peptides bind the same or different sites on clathrin NTD, with a view to developing small‐molecule inhibitors of the HDAg‐L interaction with clathrin NTD that would restrict HDV replication.

Our structural results show that peptides containing the putative CBMs of two distinct HDAg‐L sequences (HDAg‐L1_pep_ and HDAg‐L2_pep_) bind the same sites on clathrin NTD as cellular CBM peptides, binding at the clathrin box, the Royle box and (for HDAg‐L2_pep_) the arrestin box (Figure 2). However, GST pull‐down experiments performed with either purified clathrin or an oligomerized form of the NTD (His‐NTD‐NEMO) showed that these viral peptides capture NTD much less efficiently than do cellular CBM epitopes (Figure 1B,C). Previous cell‐based studies showed that mutation of HDAg‐L1 L199 to alanine severely reduced VLP production and clathrin co‐immunoprecipitation.^18^ This is consistent with our structure, as L199 forms extensive hydrophobic contacts at both the Royle and clathrin boxes (Figure 2). HDAg‐L1 mutation D203A also diminished VLP production and co‐immunoprecipitation with clathrin, but in the structures presented here this residue is consistently disordered. It is thus unclear whether this mutation directly affects binding of HDAg‐L to clathrin or has some secondary effect. Previous biochemical studies of HDAg‐L peptides binding to purified NTD also suggested that both L199 and D203 are important for the interaction.^16^, ^28^ However, these experiments should be viewed with caution as they utilized an extremely short NTD construct (residues 1‐107) that not only lacks both the Royle and arrestin box sites but also spans only the first two blades of the NTD β‐propeller and is thus highly unlikely to be correctly folded. Taken together, our results confirm that the putative CBM peptides from HDAg‐L can directly bind clathrin NTD, but do so weakly. It is therefore unclear whether these viral CBM‐like epitopes directly promote recruitment of clathrin heavy chain in vivo or whether they act synergistically with other clathrin‐adaptor proteins.

### 3.4 Degeneracy of clathrin‐binding peptide motifs

Our structural (Figure 2) and biochemical (Figure 5) studies show that 2 distinct sequence motifs can bind the arrestin box of NTD: the arrestin‐box motif ([LI][LI]GxL) and the CBM (LΦxΦ[DE]). Further, Figure 6B shows that, when presented as linear peptides, either the β2 adaptin CBM motif (GST‐AP2CBM) or an extended arrestin‐box motif (GST‐AP2arrS, where the arrestin‐box motif is followed by a leucine residue) bind the arrestin box more strongly than does the arrestin‐box motif alone (GST‐AP2arrL). This experiment also suggests that the β2 adaptin arrestin‐box motif is capable of binding the clathrin or Royle boxes (compare capture of wild‐type vs Q98A + F91K + F9W His‐NTD‐NEMO), despite this arrestin‐box motif (LLGDL) not conforming to the CBM consensus sequence. Similarly, we note that the sequences capable of binding the Royle box in crystallo are also rather degenerate, precluding the identification of a consensus binding sequence. Together, this suggests that the model of “1 consensus binding motif per peptide‐binding site on clathrin NTD” might need revisiting, as the binding of these short peptides to the NTD surface is degenerate and may depend on the structural context in which the peptides are presented.

### 3.5 Dynamics of association between clathrin terminal domain and adaptor peptides in coated pits

The results presented in this study underline the dynamic nature of clathrin:adaptor interactions and suggest that each clathrin terminal domain is capable of simultaneously binding multiple adaptors, even those containing only CBM (LΦxΦ[DE]) or arrestin‐box ([LI][LI]GxL) motifs. Individually, these interactions are of low affinity.^4^, ^15^ Proteins typically bind to both specific and non‐specific sites with similar association rates (*k*_on_), with differential affinity conferred by differing rates of dissociation (*k*_off_).^29^ Weak bimolecular interactions in the micromolar affinity range, such as those between clathrin and its adaptors, typically correspond to dissociation rates of about 1 second or a half‐time of dissociation of ~0.7 seconds.^29^ This is significantly shorter than the timescale of productive clathrin‐coated pit assembly and deformation, which occurs over the course of ~90 seconds.^30^ Thus, we would expect adaptors to display rapid cycles of binding to and dissociation from individual binding sites on clathrin, allowing recruitment of a multitude of different adaptor molecules to any given clathrin terminal domain. It is also possible that plasticity in clathrin motif binding allows individual adaptors harbouring multiple clathrin‐interaction motifs, such as epsin,^22^ to bind multiple sites on the clathrin N‐terminal domain simultaneously, thereby increasing their apparent affinity. However, as we are not yet able to define a consensus binding sequence for the Royle box, and considering the degenerate sequence requirements for binding at the clathrin or arrestin boxes, it is unclear how frequently clathrin adaptors might be able to employ such a mode of binding.

In summary, we have shown that cellular CBM peptides bind degenerately to multiple sites on clathrin, we define a set of NTD mutations at each of the 4 peptide binding sites that do not disrupt the NTD fold, and show in biochemical assays that multiple sites contribute to binding of NTD by cellular clathrin‐binding peptides. In addition, we find that viral CBM peptides bind the same sites on NTD as cellular peptides, albeit much more weakly. Finally, we present the first structural characterization of the Royle box, the fourth and final functional peptide binding site on the clathrin NTD.

## 4 MATERIALS AND METHODS

### 4.1 Constructs and mutagenesis

Wild‐type bovine clathrin heavy chain N‐terminal domain (1‐363) (NTD) with an N‐terminal glutathione S‐transferase (GST) purification tag and thrombin cleavage site was used as described previously.^11^ For binding assays, an oligomeric construct was designed by fusing clathrin NTD (1‐363) to the NF‐κB essential modulator (NEMO) oligomerization domain (246‐365) and cloning into pET‐28(a) to add an N‐terminal His_6_ purification tag (His‐NTD‐NEMO). Mutated constructs encoding His‐NTD‐NEMO with single amino acid substitutions at residues F9, E11, Q14, Q16, N17, Q89, F91, Q152, I154, W164, L183 and Q192 were generated by QuikChange site‐directed mutagenesis (Agilent) and introduction of the desired mutations was confirmed by Sanger sequencing. Clathrin‐binding motifs were cloned into pOPT3G,^31^ encoding GST followed by a human rhinovirus 3C protease cleavage site and the peptide of interest, by ligation of phosphorylated annealed oligonucleotide primers as follows: GST‐HDAg‐L1, residues 195‐214 of hepatitis D virus large antigen (HDAg‐L) clade I (UniProt P0C6L6); GST‐HDAg‐L2, residues 194‐213 of HDAg‐L clade II (UniProt A4ZNG7); GST‐AP2CBM, residues 629‐640 of the β2 adaptin subunit of human AP2 (UniProt P63010); GST‐Wbox, residues 379‐388 of human amphiphysin I (UniProt P49418); GST‐AmphCBM, residues 349‐358 of human amphiphysin I (Uniprot P49418). Two constructs containing the arrestin‐box motif (LLGDL) of the β2 adaptin subunit of human AP2 (UniProt P63010) were generated the in the same manner: GST‐AP2arrS, residues 623‐632 and GST‐AP2arrL, residues 623‐631 followed by the sequence “ASS” that corresponds to the residues C‐terminal to the LLGDL arrestin‐box motif of arrestin2L.^13^ An additional construct (GST‐Amph4T1) encoding residues 349‐356 of rat amphiphysin I (UniProt O08838; rat and human amphiphysin I residues 349‐356 are identical), inserted into pGEX‐4T1 after EcoRI/XhoI digestion and thus encoding 7 additional amino acids (LERPHRD) C‐terminal to the amphiphysin sequence, was described previously.^22^

### 4.2 Protein expression and purification

A clathrin‐coated vesicle fraction was isolated from pig brains essentially as described.^32^ Coat proteins were stripped from the vesicles^33^ and clathrin was purified from the coat protein mixture by gel filtration in 0.5 M Tris pH 7.0, 1 mM DTT (Superose 6, GE Healthcare), dialyzed into 10 mM Tris pH 8.0 and stored at 4°C. All other proteins were expressed in *E. coli* strains BL21(DE3) pLysS (GST‐tagged constructs) or B834(DE3) (wild‐type and mutant His‐NTD‐NEMO). Bacteria were grown in 2×TY medium with appropriate selection antibiotics to an optical density (OD)_600_ of 0.8‐1.0, the temperature was reduced to 22°C and protein expression was induced by addition of 0.2 mM isopropyl β‐d‐thiogalactopyranoside. After 12‐18 h cells were harvested by centrifugation (6000 × g, 15 min, 4°C) and stored at −80°C.

Bacterial pellets containing GST‐NTD were resuspended in lysis buffer (20 mM Tris pH 7.5, 200 mM NaCl, 0.05% TWEEN‐20, 0.5 mM MgCl_2_, 1.4 mM β‐mercaptoethanol) supplemented with 200‐400 U bovine DNase I (Sigma) and 200 μL ethylenediaminetetraacetic acid (EDTA)‐free protease inhibitor cocktail (Sigma). Cells were lysed at 24 kpsi using a cell disruptor (Constant Systems) and the lysate was cleared by centrifugation (40 000*g*, 30 min, 4°C). Cleared lysate was incubated with glutathione Sepharose 4B (GE Healthcare) for 60 minutes at 4°C, the beads were washed (20 mM Tris pH 7.5, 200 mM NaCl, 1 mM DTT), equilibrated in thrombin cleavage buffer (20 mM Tris pH 7.5, 200 mM NaCl, 1 mM CaCl_2_), and the GST tag removed by overnight incubation at room temperature with thrombin (125 U, Serva). Following incubation with fresh glutathione resin to capture liberated GST and uncleaved GST‐NTD fusion, NTD was further purified using a HiLoad Superdex 200 size exclusion chromatography column (GE Healthcare) equilibrated in 10 mM Tris pH 7.5, 50 mM NaCl, 4 mM DTT. Following concentration, small aliquots (20‐100 μL) of purified NTD were snap‐frozen in liquid nitrogen and stored at −80°C.^34^ For other GST‐tagged proteins, cell pellets were resuspended in lysis buffer (20 mM HEPES pH 7.5, 300 mM NaCl, 0.05% TWEEN‐20, 0.5 mM MgCl_2_, 1.4 mM β‐mercaptoethanol) supplemented with DNase I and protease inhibitors as above. Cells were lysed, and lysates were clarified and incubated with glutathione resin as above. The glutathione resin was washed (20 mM HEPES pH 7.5, 300 mM NaCl, 1 mM DTT) and bound proteins were eluted using wash buffer supplemented with 25 mM reduced glutathione. Following size exclusion chromatography using HiLoad Superdex 75 or 200 columns (GE Healthcare) equilibrated in 20 mM HEPES pH 7.5, 200 mM NaCl, 1 mM DTT, fusion proteins were mixed 1:1 with 100% (v/v) glycerol and stored at −20°C.

For wild‐type and mutant His‐NTD‐NEMO, cell pellets were resuspended in lysis buffer (20 mM Tris pH 7.5, 500 mM NaCl, 0.05% TWEEN‐20, 0.5 mM MgCl_2_, 1.4 mM β‐mercaptoethanol) supplemented with 2‐20 mg hen egg white lysozyme (Sigma), 400 U bovine DNase I (Sigma) and 200 μL EDTA‐free protease inhibitor cocktail (Sigma). The cells were lysed and the lysate clarified as described above. The cleared lysate was applied to a 1 mL HisTrap excel Ni affinity column (GE Healthcare), the column was washed (20 mM Tris pH 7.5, 500 mM NaCl, 12.5 mM imidazole pH 7.5) and the protein eluted (20 mM Tris pH 7.5, 500 mM NaCl, 250 mM imidazole pH 7.5). The Ni affinity column eluate was injected onto a HiLoad Superdex 200 column (GE Healthcare) equilibrated in 20 mM Tris pH 7.5, 200 mM NaCl, 1 mM DTT and eluted in distinct peaks, which were collected and concentrated separately to yield His‐NTD‐NEMO and His‐NTD (Figure S1), which were both were snap‐frozen in liquid nitrogen as described above.

### 4.3 Crystallization, data collection, structure determination and analysis

Crystals were grown at 20°C by sitting drop vapour diffusion. Clathrin NTD (1‐363) was co‐crystallized in complex with the following peptides (peptide sequences are listed with residues not present in the GST‐tagged constructs underlined): Amph4T1_pep_ (ETLLDLDFLE); AmphCBM_pep_ (ETLLDLDFDP); AP2CBM_pep_ (CGDLLNLDLG); HDAg‐L1_pep_ (SDILFPADS); HDAg‐L2_pep_ (SPRLPLLES); AmphCBMlong_pep_ (ETLLDLDFDPFK). Peptides were purchased from Genscript (Amph4T1_pep_, AmphCBM_pep_ and AmphCBMlong_pep_) or Designer Bioscience (AP2CBM_pep_, HDAg‐L1_pep_ and HDAg‐L2_pep_). All peptides were prepared as 10 mM stock solutions in 10 mM Tris pH 7.5, 50 mM NaCl, 4 mM DTT and stored at −20°C. NTD was mixed 2:1 with peptide to give final concentrations of 14 mg/mL NTD and 3.4 mM peptide for all crystallization experiments except NTD:HDAg‐L2_pep_, where 20 mg/mL NTD and 3.6 mM peptide was used. Crystals for structure determination were obtained under the following conditions (P and R indicate peptide:protein and reservoir volumes in sitting drops, respectively): NTD:Amph4T1_pep_, 1 μL P plus 1 μL R equilibrated against a 200 μL reservoir of 1.1 M sodium malonate pH 8.0 (Hampton Research); NTD:AmphCBM_pep_, 1 μL P plus 2 μL R equilibrated against a 200 μL reservoir of 0.85 M sodium malonate pH 7.5; NTD:AP2CBM_pep_, 1 μL P plus 2 μL R equilibrated against a 200 μL reservoir of 0.94 M sodium malonate pH 6.7; NTD:HDAg‐L1_pep_, 400 nL P plus 200 nL R equilibrated against a 80 μL reservoir of 1.21 M sodium malonate pH 7.0; NTD:HDAg‐L2_pep_, 200 nL P plus 400 nL R equilibrated against a 80 μL reservoir of 1.75 M sodium malonate pH 7.0; NTD:AmphCBMlong_pep_, 1 μL P plus 2 μL R (1.04 M sodium malonate pH 7.1, 0.2 M sodium perchlorate [Jena Bioscience]) equilibrated against a 200 μL reservoir of 1.15 M sodium malonate pH 7.1. All crystals were cryoprotected by rapid transfer into a drop comprising 55% reservoir solution, 25% (v/v) glycerol and 20% (v/v) 10 mM peptide stock solution, the peptide being added to prevent dissociation from NTD of bound peptides. Crystals were then immediately flash‐cooled in liquid nitrogen and stored in liquid nitrogen.

Diffraction data were recorded at 100 K on Diamond Light Source beamlines I02 (NTD:HDAg‐L1_pep_, NTD:HDAg‐L2_pep_ and NTD:AmphCBMlong_pep_) and I04‐1 (NTD:Amph4T1_pep_, NTD:AmphCBM_pep_ and NTD:AP2CBM_pep_). Data were processed using XDS, XSCALE and Aimless (NTD:Amph4T1_pep_, NTD:HDAg‐L1_pep_ and NTD:AmphCBMlong_pep_), or DIALS and Aimless (NTD:AmphCBM_pep_ and NTD:AP2CBM_pep_), as implemented by the xia2 automated processing pipeline,^35^, ^36^, ^37^, ^38^, ^39^, ^40^, ^41^ or using iMOSFLM^42^ and Aimless interactively (NTD:HDAg‐L2_pep_). The structures of the NTD:HDAg‐L1_pep_, NTD:HDAg‐L2_pep_, NTD:Amph4T1_pep_, NTD:AmphCBM_pep_ and NTD:AmphCBMlong_pep_ complexes were solved by isomorphous replacement in REFMAC5^43^, ^44^ using a high‐resolution ligand‐free model of NTD (PDB 1C9I)^10^ as a starting model. The structure of the NTD:AP2CBM_pep_ complex was solved by molecular replacement with a single chain of NTD (PDB 1C9I, chain A) as a search model using Phaser.^45^ Manual model building was performed using COOT^46^ and the models were refined using REFMAC5. In all structures, the peptides were modelled after initial improvement of the peptide‐free structure. The geometric quality of the models was improved by consulting the validation tools in COOT as well as the programmes MolProbity^47^ and WHAT_CHECK.^48^ Structure factors and final refined models have been deposited with the Protein Data Bank as listed in Table 1 and Table S2. Feature‐enhanced maps, which have reduced model bias and optimized scaling to ease comparison of strong and weak features, were calculated using phenix.fem.^24^, ^49^ Evolutionary conservation of amino acids was estimated using ConSurf^50^ with default parameters and chain A of the refined NTD:Amph4T1_pep_ structure as an input model. PyMOL (Schrodinger) was used to generate molecular graphics and figures were assembled using Inkscape (https://inkscape.org/).

### 4.4 Capture (GST pull‐down) assays and immunoblotting

All steps of the clathrin or His‐NTD‐NEMO capture assays were performed at 4°C using a previously published protocol^11^ adapted to enable detection of the very weak interactions investigated in this work. A total of 40 μL of glutathione sepharose 4B beads (GE Healthcare) pre‐equilibrated in assay buffer (25 mM HEPES‐KOH, 125 mM potassium acetate, 5 mM magnesium acetate, 2 mM EDTA, 2 mM EGTA, 1 mM DTT, pH 7.2) were incubated for 2 h with 20 µg (for purified clathrin pull‐down experiments) or 500 µg (for recombinant NTD pull‐downs) of GST or GST fusion proteins in assay buffer to a final volume of 400 μL. Non‐immobilized bait protein was removed following centrifugation (10 000*g*, 2 min) and the resin was washed thrice with assay buffer. The protein‐loaded resin was then incubated with 300 μL of 0.1 mg/mL His‐NTD‐NEMO or His‐NTD, or 0.4 μM purified clathrin, for 2 hours. Following centrifugation, supernatants containing uncaptured protein were retained for analysis and the resins were washed 4× with phosphate‐buffered saline. After the final wash, the resin pellet was resuspended in 80 μL SDS‐PAGE buffer and the samples were eluted by boiling for 5 min at 95°C. Input and supernatant samples were prepared in SDS‐PAGE buffer. Samples (0.33% of the prey input samples, 11.25% of the eluted pellet samples, 0.6% of the supernatant kept after the prey incubation) were separated by SDS‐PAGE and transferred to nitrocellulose membranes before immunoblotting using a mouse anti‐clathrin N‐terminal domain primary antibody (ab11221, Abcam) and fluorescently labelled goat anti‐mouse secondary antibody (925‐32210, LI‐COR). Dried membranes were visualized using an Odyssey scanner (LI‐COR).

### 4.5 Biophysical assays: CD spectroscopy, differential scanning fluorimetry and multi‐angle light scattering

Circular dichroism spectra were recorded on a Jasco J‐810 spectropolarimeter at 20°C. Protein samples were diluted to 1 mg/mL in 50 mM phosphate buffer, pH 7.4. A total of 8 spectra per sample were recorded (50 nm/min, 1 nm bandwidth, 260‐190 nm), averaged, and smoothed (Savitzky and Golay method, second order smoothing, 5 nm sliding window) using Prism 5 (GraphPad Software).

Differential scanning fluorimetry experiments to determine the melting temperature (T_M_) of wild‐type or mutant His‐NTD were performed using a MiniOpticon real‐time PCR system (BioRad) with 10× SYPRO Orange dye (Molecular Probes) or Viia7 real‐time PCR system (Applied Biosystems) using 1× Protein Thermal Shift dye (Applied Biosystems). Multiple experiments confirmed that the difference between the T_M_ of wild‐type His‐NTD and mutants (T_M_[wt]‐T_M_[mutant]) is measured consistently using either platform. In all experiments, assay buffer (20 mM HEPES‐KOH, 120 mM potassium acetate, pH 7.5) was mixed with dye stock solution and protein solution in an 8:1:1 ratio, to give 10 ng protein in a final volume of 50 μL (MiniOpticon) or 2 ng protein in a final volume of 20 μL (Viia7). Samples (measured in triplicate) were heated from 20°C to 90°C at 1 K/minutes (MiniOpticon) or 25 to 95°C at 1 K/20 seconds (Viia7) and fluorescence was monitored at 1 K increments. Curve fitting, melting temperature calculations and plotting were performed using MATLAB (MathWorks).

Multi‐angle light scattering (MALS) experiments^51^ were performed at 22°C using a Superdex 200 10/300 gel filtration column (GE Healthcare) equilibrated in 20 mM Tris pH 7.5, 200 mM NaCl, 1 mM DTT. Samples (100 μL) were injected at a flow rate of 0.5 mL/min and size exclusion was followed by inline measurement of static light scattering (DAWN 8+, Wyatt Technology) and differential refractive index (Optilab T‐rEX, Wyatt Technology). The data were analysed using Astra6 (Wyatt Technology).

## Supporting information

Editorial Process

**TABLE S1**. Average atomic displacement parameters (ADPs) of N‐terminal domain (NTD) peptide‐binding residues and of bound peptides. In crystal structures at near‐atomic resolution the ADP of an atom is an indication of its degree of order. Peptide‐binding residues were defined as any NTD residue within 5 Å of a bound peptide, not including molecules related by crystallographic symmetry. Average isotropic ADPs for were calculated using phenix.pdbtools^25^. ΔADP is the difference between mean ADPs of the peptide and the residues to which it binds.
**TABLE S2**. Crystallographic data collection and refinement of N‐terminal domain (NTD) co‐crystallized with AmphCBMlong_pep_. AmphCBMlong_pep_ corresponds in sequence to residues 349‐360 of human amphiphysin I (UniProt P49418). Values for the highest resolution shell are shown in parentheses.
**FIGURE S1**. His‐N‐terminal domain‐NF‐κB essential modulator (NTD‐NEMO) forms oligomers and is captured more efficiently than His‐NTD by glutathione S‐transferase (GST)‐tagged clathrin‐binding peptides (A) Capture (“GST pull‐down”) of His‐NTD‐NEMO and His‐NTD by GST‐AP2CBM. His‐NTD was produced by taking advantage of a proteolytic cleavage event that occurred during the purification of wild‐type and mutant His‐NTD‐NEMO. During gel filtration chromatography a significant amount of protein eluted at a volume consistent with it lacking the NEMO domain. As this protein reacted with an anti‐NTD antibody and its experimental mass was as expected for His‐NTD alone (B) we assumed it to be His‐NTD alone, the NEMO oligomerization domain having been liberated by proteolysis during the bacterial expression or subsequent lysis and affinity purification. Glutathione sepharose beads loaded with GST‐AP2CBM bait protein were incubated with His‐NTD‐NEMO or His‐NTD, washed, and the beads were collected. The input NTD samples (I), supernatant following prey incubation (S) and bound protein sample following washing (P) were subjected to SDS‐PAGE and immunoblotting (WB) using an antibody that recognizes clathrin NTD (αNTD). His‐NTD‐NEMO is more readily captured than His‐NTD in this assay. B, Determination of the mass of His‐NTD (green) and His‐NTD‐NEMO (purple) by size‐exclusion chromatography with inline multi‐angle light scattering (SEC‐MALS). The elution profiles of each protein, monitored using the solvent differential refractive index (dRI), are shown as dashed curves. Weight‐averaged molar masses, determined directly from the dRI and light scattering of the samples^24^, are shown as solid lines across the elution profiles. The expected molar masses for a His‐NTD monomer and a His‐NTD‐NEMO monomer are shown as dotted grey lines. (C) Immunoblot analysis of the 2 main elution peaks observed during SEC‐MALS of His‐NTD‐NEMO. The weight‐averaged molar mass is shown as a line across the elution profile (upper panel, purple lines). Fractions collected throughout the experiment (grey ticks on horizontal axis) were subjected to SDS‐PAGE and immunoblotting as in (A). Only the larger peak, eluting between 12 and 14 mL, is recognized by the anti‐clathrin NTD antibody and thus the smaller peak, eluting between 11 and 12 mL, is presumed to be a co‐purified contaminant. D, Concentration‐dependent oligomerization of His‐NTD‐NEMO. SEC‐MALS was performed using His‐NTD‐NEMO injected at 6 different concentrations. While the weight‐averaged molar mass of the eluted protein (solid lines) matches the expected molar mass of monomeric His‐NTD‐NEMO (grey dotted line) at low concentrations of injected His‐NTD‐NEMO, the protein exhibits lower elution volumes (monitored using dRI, dashed line) and increased weight‐averaged molar mass when the concentration of injected protein increases. This is consistent with homo‐oligomerization of His‐NTD‐NEMO, presumably mediated by the NEMO oligomerization domain^23^.
**FIGURE S2**. AmphCBMlong_pep_ binds NTD at the clathrin and arrestin boxes but not the Royle box. The β‐propeller fold of clathrin NTD (grey ribbons) is shown with numbers enumerating the 7 β‐stranded blades. Spheres represent peptides bound at the 4 peptide‐interaction sites on N‐terminal domain (NTD). Insets show unbiased *F*_O_‐*F*_C_ electron density (3 σ), calculated before the addition of peptide residues to the structural model, that is consistent with binding of AmphCBMlong_pep_ at the clathrin and arrestin boxes but not at the Royle box. The final refined model of AmphCBMlong_pep_ (sticks, carbon atoms cyan) bound at the clathrin and arrestin sites is shown with selected NTD side chain atoms also displayed (sticks, carbon atoms grey).
**FIGURE S3**. Circular dichroism of wild‐type and mutant clathrin N‐terminal domain (NTD). Circular dichroism (CD) spectra of wild‐type (black) or mutant (coloured) His‐NTD. The spectra are consistent with His‐NTD having a predominantly β‐sheet composition, as expected from the clathrin NTD crystal structure. None of the His‐NTD mutants have significantly different CD spectra, consistent with them all having secondary structure content similar to wild‐type His‐NTD.
