# Supplementary material for: Cellular and viral peptides bind multiple sites on the N‐terminal domain of clathrin
Source: Traffic. 2016 Dec 14;18(1):44–57. doi: 10.1111/tra.12457 (PMC5182127; doi:10.1111/tra.12457)
Supplement: Supplementary file 2 — TABLE S1. Average atomic displacement parameters (ADPs) of N‐terminal domain (NTD) peptide‐binding residues and of bound peptides. In crystal structures at near‐atomic resolution the ADP of an atom is an indication of its degree of order. Peptide‐binding residues were defined as any NTD residue within 5 Å of a bound peptide, not including molecules related by crystallographic symmetry. Average isotropic ADPs for were calculated using phenix.pdbtools25. ΔADP is the difference between mean ADPs of the peptide and the residues to which it binds. TABLE S2. Crystallographic data collection and refinement of N‐terminal domain (NTD) co‐crystallized with AmphCBMlongpep. AmphCBMlongpep corresponds in sequence to residues 349‐360 of human amphiphysin I (UniProt P49418). Values for the highest resolution shell are shown in parentheses. FIGURE S1. His‐N‐terminal domain‐NF‐κB essential modulator (NTD‐NEMO) forms oligomers and is captured more efficiently than His‐NTD by glutathione S‐transferase (GST)‐tagged clathrin‐binding peptides (A) Capture (“GST pull‐down”) of His‐NTD‐NEMO and His‐NTD by GST‐AP2CBM. His‐NTD was produced by taking advantage of a proteolytic cleavage event that occurred during the purification of wild‐type and mutant His‐NTD‐NEMO. During gel filtration chromatography a significant amount of protein eluted at a volume consistent with it lacking the NEMO domain. As this protein reacted with an anti‐NTD antibody and its experimental mass was as expected for His‐NTD alone (B) we assumed it to be His‐NTD alone, the NEMO oligomerization domain having been liberated by proteolysis during the bacterial expression or subsequent lysis and affinity purification. Glutathione sepharose beads loaded with GST‐AP2CBM bait protein were incubated with His‐NTD‐NEMO or His‐NTD, washed, and the beads were collected. The input NTD samples (I), supernatant following prey incubation (S) and bound protein sample following washing (P) were subjected to SDS‐PAGE and immunoblotting (WB [file TRA-18-44-s001.docx]

**Cellular and viral peptides bind multiple sites on the N-terminal domain of clathrin**

Julia Muenzner^1^, Linton M. Traub^2^, Bernard T. Kelly^3^*, Stephen C. Graham^1^*

^1^Department of Pathology, University of Cambridge, Tennis Court Road, Cambridge CB2 1QP, UK

^2^Department of Cell Biology, University of Pittsburgh School of Medicine, Pittsburgh, PA 15261, USA

^3^Cambridge Institute for Medical Research, Department of Clinical Biochemistry, University of Cambridge, Hills Road, Cambridge CB2 0XY, UK

*Corresponding authors: Bernard T. Kelly, [btk1000@cam.ac.uk](mailto:btk1000@cam.ac.uk), and Stephen C. Graham, [scg34@cam.ac.uk](mailto:scg34@cam.ac.uk)

**Table S1: Average atomic displacement parameters (ADPs) of NTD peptide-binding residues and of bound peptides.** In crystal structures at near-atomic resolution the ADP of an atom is an indication of its degree of order. Peptide-binding residues were defined as any NTD residue within 5 Å of a bound peptide, not including molecules related by crystallographic symmetry. Average isotropic ADPs for were calculated using phenix.pdbtools. ΔADP is the difference between mean ADPs of the peptide and the residues to which it binds.

| **Structure** | **Chain** | **Clathrin box** | | | **Arrestin box** | | | **Royle box** | | |
| --- | --- | --- | --- | --- | --- | --- | --- | --- | --- | --- |
|  |  | **Peptide** | **NTD** | **ΔADP** | **Peptide** | **NTD** | **ΔADP** | **Peptide** | **NTD** | **ΔADP** |
| **AP2CBM_pep_** | A | 48.4 | 33.2 | 15.2 | 57.9 | 29.2 | 28.7 | – | – | – |
| **AmphCBM_pep_** | A | 34.7 | 19.6 | 15.1 | 53.6 | 20.9 | 32.7 | – | – | – |
|  | B | 38.3 | 22.2 | 16.1 | 49.1 | 18.2 | 30.9 | – | – | – |
| **Amph4T1_pep_** | A | 31.0 | 21.0 | 10.0 | 50.8 | 23.5 | 27.3 | 53.5 | 32.7 | 20.8 |
|  | B | 36.8 | 24.2 | 12.6 | 63.8 | 22.6 | 41.2 | 56.2 | 31.7 | 24.5 |
| **HDAg-L1_pep_** | A | 57.5 | 37.4 | 20.1 | – | – | – | 76.0 | 45.3 | 30.6 |
|  | B | 75.4 | 46.5 | 29.0 | – | – | – | 72.8 | 46.7 | 26.1 |
| **HDAg-L2_pep_** | A | 42.3 | 28.9 | 13.4 | 64.8 | 28.1 | 36.7 | 73.7 | 38.4 | 35.3 |
|  | B | 49.6 | 34.8 | 14.8 | 55.8 | 26.1 | 29.8 | 75.5 | 39.7 | 35.9 |

Table S2: Crystallographic data collection and refinement of NTD co-crystallised with AmphCBMlong_pep_. AmphCBMlong_pep_ corresponds in sequence to residues 349–360 of human amphiphysin I (UniProt P49418). Values for the highest resolution shell are shown in parentheses.

|  | **NTD:AmphCBMlong_pep_** |
| --- | --- |
| **Data collection** |  |
| Space group | *C*2 |
| Cell dimensions |  |
| *a*, *b*, *c* (Å) | 138.1, 131.2, 77.8 |
| α, β, γ (°) | 90.0, 115.4, 90.0 |
| Resolution (Å) | 39.7–1.8 (1.89–1.84) |
| *R*_merge_ | 0.084 (1.232) |
| < I/σI> | 12.8 (1.5) |
| CC_1/2_ | 0.999 (0.528) |
| Completeness (%) | 99.9 (99.5) |
| Redundancy | 6.8 (6.2) |
| **Refinement** |  |
| Resolution (Å) | 39.7–1.8 (1.89–1.84) |
| No. of reflections (R_work_/R_free_) | 102,809/5312 |
| R_work_/R_free_ | 0.162/0.182 |
| Ramachandran favored regions (%) | 98.4 |
| Ramachandran outliers (%) | 0.0 |
| No. of atoms |  |
| Protein | 5772 |
| Glycerol | 6 |
| Peptide ligands | 336 |
| Water | 785 |
| B-factors |  |
| Protein | 31.9 |
| Glycerol | 27.1 |
| Peptide ligands | 57.9 |
| Water | 46.4 |
| r.m.s. deviations |  |
| Bond lengths (Å) | 0.012 |
| Bond angles (°) | 1.549 |
| PDB ID | 5M61 |


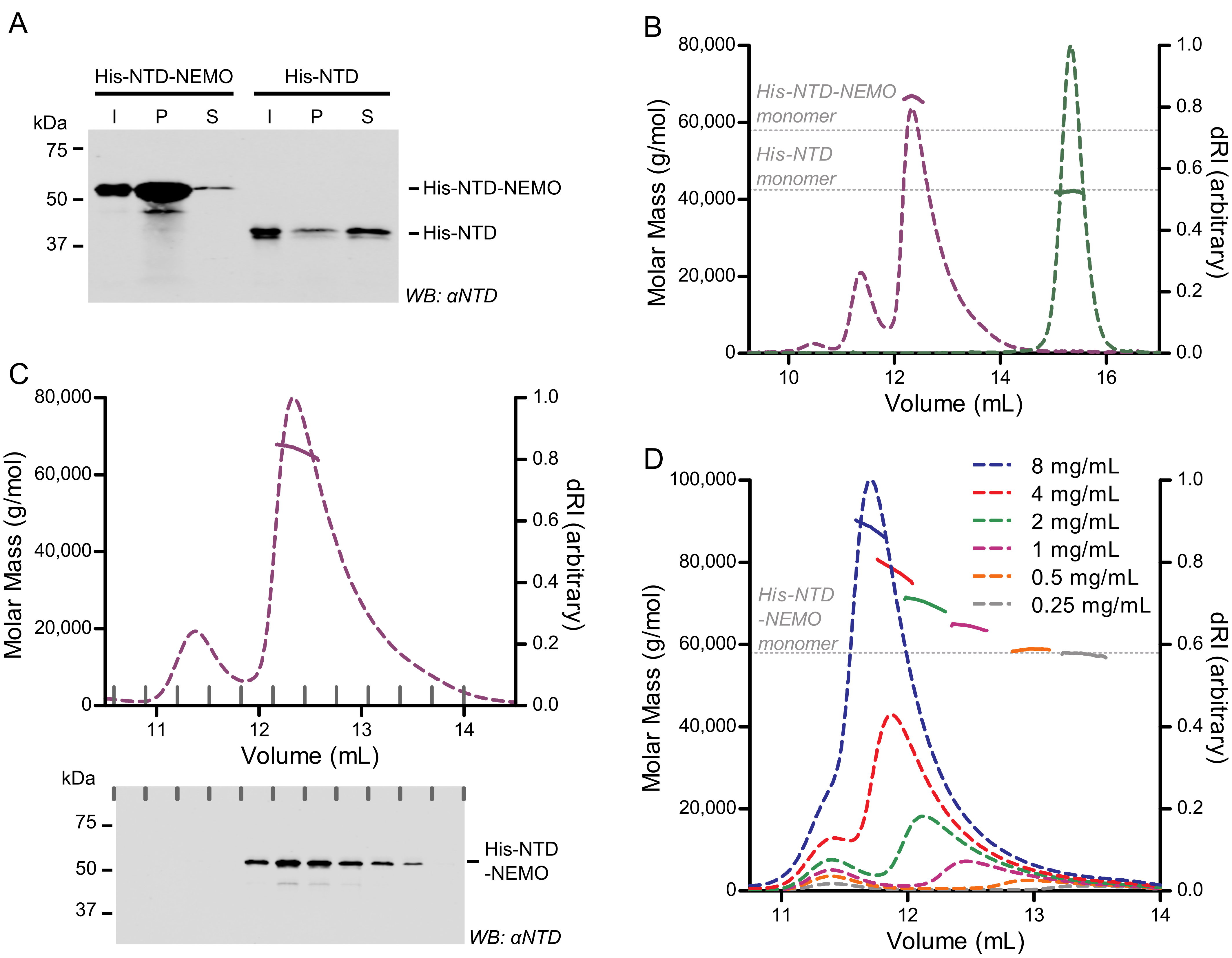


**Figure S1: His-NTD-NEMO forms oligomers and is captured more efficiently than His-NTD by GST-tagged clathrin-binding peptides** (A) Capture (“GST pull-down”) of His-NTD-NEMO and His-NTD by GST-AP2CBM. His-NTD was produced by taking advantage of a proteolytic cleavage event that occurred during the purification of wild-type and mutant His-NTD-NEMO. During gel filtration chromatography a significant amount of protein eluted at a volume consistent with it lacking the NEMO domain. As this protein reacted with an anti-NTD antibody and its experimental mass was as expected for His-NTD alone (B) we assumed it to be His-NTD alone, the NEMO oligomerisation domain having been liberated by proteolysis during the bacterial expression or subsequent lysis and affinity purification. Glutathione sepharose beads loaded with GST-AP2CBM bait protein were incubated with His-NTD-NEMO or His-NTD, washed, and the beads were collected. The input NTD samples (I), supernatant following prey incubation (S) and bound protein sample following washing (P) were subjected to SDS-PAGE and immunoblotting (WB) using an antibody that recognizes clathrin NTD (αNTD). His-NTD-NEMO is more readily captured than His-NTD in this assay. (B) Determination of the mass of His-NTD (green) and His-NTD-NEMO (purple) by size-exclusion chromatography with inline multi-angle light scattering (SEC-MALS). The elution profiles of each protein, monitored using the solvent differential refractive index (dRI), are shown as dashed curves. Weight-averaged molar masses, determined directly from the dRI and light scattering of the samples, are shown as solid lines across the elution profiles. The expected molar masses for a His-NTD monomer and a His-NTD-NEMO monomer are shown as dotted grey lines. (C) Immunoblot analysis of the two main elution peaks observed during SEC-MALS of His-NTD-NEMO. The weight-averaged molar mass is shown as a line across the elution profile (upper panel, purple lines). Fractions collected throughout the experiment (grey ticks on horizontal axis) were subjected to SDS-PAGE and immunoblotting as in (A). Only the larger peak, eluting between 12–14 mL, is recognised by the anti-clathrin NTD antibody and thus the smaller peak, eluting between 11–12 mL, is presumed to be a co-purified contaminant. (D) Concentration-dependent oligomerisation of His-NTD-NEMO. SEC-MALS was performed using His-NTD-NEMO injected at six different concentrations. While the weight-averaged molar mass of the eluted protein (solid lines) matches the expected molar mass of monomeric His-NTD-NEMO (grey dotted line) at low concentrations of injected His-NTD-NEMO, the protein exhibits lower elution volumes (monitored using dRI, dashed line) and increased weight-averaged molar mass when the concentration of injected protein increases. This is consistent with homo-oligomerisation of His-NTD-NEMO, presumably mediated by the NEMO oligomerisation domain.

**
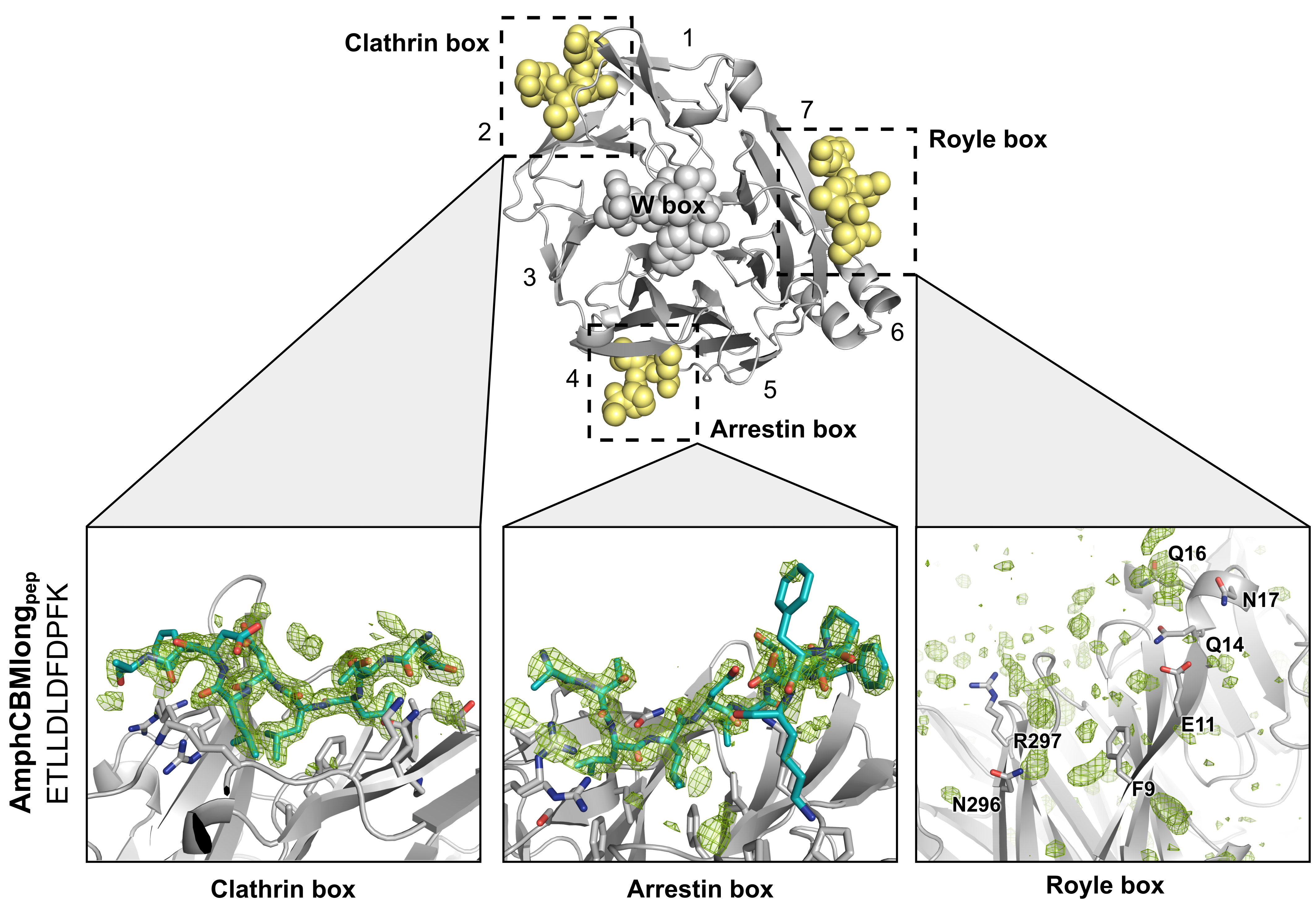
**

**Figure S2: AmphCBMlong_pep_ binds NTD at the clathrin and arrestin boxes but not the Royle box.** The β-propeller fold of clathrin NTD (grey ribbons) is shown with numbers enumerating the seven β-stranded blades. Spheres represent peptides bound at the four peptide-interaction sites on NTD. Insets show unbiased *F*_O_-*F*_C_ electron density (3 σ), calculated before the addition of peptide residues to the structural model, that is consistent with binding of AmphCBMlong_pep_ at the clathrin and arrestin boxes but not at the Royle box. The final refined model of AmphCBMlong_pep_ (sticks, carbon atoms cyan) bound at the clathrin and arrestin sites is shown with selected NTD side chain atoms also displayed (sticks, carbon atoms grey).


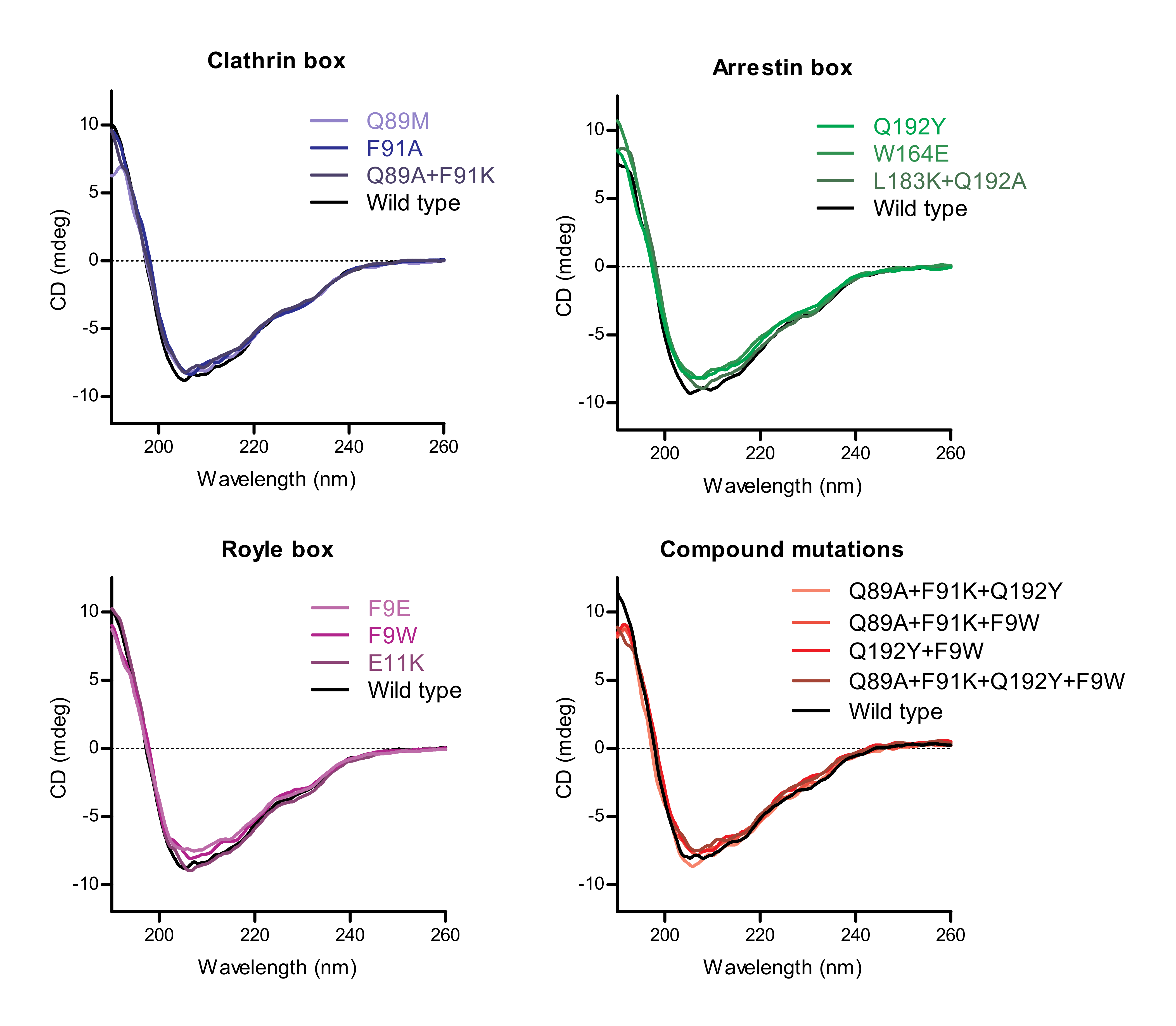


**Figure S3: Circular dichroism of wild-type and mutant clathrin NTD.** Circular dichroism (CD) spectra of wild-type (black) or mutant (coloured) His-NTD. The spectra are consistent with His-NTD having a predominantly β-sheet composition, as expected from the clathrin NTD crystal structure. None of the His-NTD mutants have significantly different CD spectra, consistent with them all having secondary structure content similar to wild-type His-NTD.
