# Supplementary material for: Cellular and viral peptides bind multiple sites on the N‐terminal domain of clathrin
Source: Traffic. 2016 Dec 14;18(1):44–57. doi: 10.1111/tra.12457 (PMC5182127; doi:10.1111/tra.12457)
Supplement: Supplementary file 1 — Editorial Process [file TRA-18-44-s002.pdf]

Title: **Cellular and viral peptides bind multiple sites on the N-terminal domain of clathrin**

Authors: Julia Muenzner, Linton M. Traub, Bernard T. Kelly, Stephen C. Graham

Article Type: Original Research

Monitoring Editor Mark Marsh
Date Submitted 28 June, 2016
Date for Decision 1 1 August 2016

Date Resubmitted 25 October 2016
Accepted 31 October 2016

Decision and Reviews

Dear Dr. Kelly and Dr. Graham,

Regarding your manuscript "Cellular and viral peptides bind multiple sites on the N-terminal domain of clathrin" submitted for consideration for publication in Traffic, I asked two colleagues who are experts in the field to review the paper and their verbatim comments are appended below. Although both reviewers point out the lack of any physiological link, you will see that both are enthusiastic about the work and recommend publication in Traffic. They do make some suggestions on additions that will clarify the conclusions, in my view you should go at least some way to addressing these points as well as incorporating the corrections indicated by reviewer 2.

Although I cannot accept your manuscript for publication at this point, I believe that you will be able to address the referees' concerns and I look forward to receiving your revised manuscript. To expedite handling when you resubmit please include a response outlining how you have addressed each of the referees' concerns.

Yours sincerely,

Mark Marsh
Associate Editor
____
Referee's Comments to the Authors

Referee: 1

Comments to the Author
In this study, the authors have solved the high resolution structures of clathrin TD in complex with various CBM peptides. Surprisingly, in addition to binding to clathrin-box sites, all CBM peptides also showed density at the arrestin box binding region . The new interaction provides a structural basis to explain the redundancy of clathrin recruitment. Moreover, several CBM peptides from amphiphysin or Hepatitis D virus large antigen were found to bind to the Royle box site. This is the first report to confirm the "Royle" binding site at the structural level. They further carry out GST pull down assays in combination with mutagenesis work to evaluate these binding surfaces.

Overall, the manuscript is well-written and the structures support the conclusion that CBM peptide can bind to multiple sites in clathrin NTD. The main limitation of the study is that it does not explore the physiological consequences. Nevertheless, the in vitro and high resolution structural studies more than cross the bar for publication in Traffic.

The authors should consider the following questions. I would encourage but not insist on carrying out further controls experiments to clarify these points.

1) In Figure 2: The unbiased density maps of HDAg-L1, HDAg-L2 in the arrestin box and Royle box are disconnected and do not resolve side chain positions unambiguously. Perhaps this would be clearer at a lower contour level? If the assignments cannot be better supported the authors should be clear about the ambiguity (Figure 4).

2) In Figure 2: there is clear evidence of CBM peptide in Royle box for Amphiphysin4T1. Since AmphiphysinCBM wasn't predicted to dock in this region, it may indicate that the artificial residues (LERPHRD) may have driven peptide binding to the Royle box. More

likely the AmphyiphysinCBM peptide is just not long enough on its own to allow it docking in Royle box and simply needs some flanking residues. This could be tested.

3) In the linker of beta2 adaptin (621 IPSQGDLLGD LLNLDLGPPV 640), the sequence contains both a CBM motif and an arrestin box motif ([LI][LI]GxL.). The authors might want to compare which motif favors binding to the arrestin box.

4) The Amphiphysin sequence also contains two signals: a clathrin box motif and a W box motif (351 LLDLDFDPFK PEVTPAGSAG VTHSPMSQTL PWDLWTTSTD 390). The linker in between is about 20 aa. Miele, et al 2004 showed that the longer peptide in amphiphysin containing these two motifs
enhances the binding of clathrin NTD by 6 folds (from 28uM to <5uM) (ref).
The authors may wish to test the longer peptide to check the binding preference.

Referee: 2

Comments to the Author
Main points.

This detailed structural/biochemistry paper analyses the binding of short, linear 'clathrin box motif' (CBM) peptides to the beta-propeller of the clathrin N-terminal domain (NTD). Perhaps the most significant finding is that NTD binding peptides of beta-2 adaptin, amphiphysin and HDAg-L2 bind to both the clathrin and arrestin box sites (in contrast with previous findings). The authors also defined a fourth peptide binding site on the NTD which they called the 'Royle box'.

This is a clear, straightforward structural paper and I have little to add other than some minor suggestions.

First, it would be helpful to have a broader context (perhaps in the discussion) to frame the detailed information presented. Presumably these different proteins can't all bind the NTD at the same time. How do the authors envisage that the different cellular binding partners 'share time' on the NTD? Is it an ordered, sequential series of binding interactions or some kind of competitive free-for-all?

Second, how confident are the authors in the 'Royle box' as a 'consensus' binding site given that similar peptides bind in different orientations and that binding does not involve the consensus CBM motif? This is especially true since given the high concentrations of peptides used in the study (~3mM(+)).

Minor points:

Abstract – Sentence starting 'Surprisingly, with each peptide …'. The sentence doesn't scan, presumably ',' should be replaced by 'and' to read: 'Surprisingly, with each peptide we observe simultaneous peptide binding at multiple sites on the NTD and viral peptides binding to the same sites as cellular peptides.

I think 'Fig 5E' is cited before 'Fig 5D'.

Author Rebuttal

Dear Prof Marsh,

Please find attached a revised version of our manuscript entitled "Cellular and viral peptides bind multiple sites on the N-terminal domain of clathrin" (Manuscript ID TRA-16-0533).

We thank the reviewers for their supportive comments and suggestions for informative further experiments. We have addressed their concerns by performing significant further experiments, including the elucidation of a new crystal structure between clathrin NTD and an extended peptide containing the human amphiphysin I clathrin-box motif. A point-by-point response to the reviewers' comments is attached.

We hope that you will now find our revised article suitable for publication in Traffic.

Yours sincerely,

Stephen Graham and Bernie Kelly

Editor's comments

Regarding your manuscript "Cellular and viral peptides bind multiple sites on the N-terminal domain of clathrin" submitted for consideration for publication in Traffic, I asked two colleagues who are experts in the field to review the paper and their verbatim comments are appended below. Although both reviewers point out the lack of any physiological link, you will see that both are enthusiastic about the work and recommend publication in Traffic. They do make some suggestions on additions that will clarify the

conclusions, in my view you should go at least some way to addressing these points as well as incorporating the corrections indicated by reviewer 2.

Although I cannot accept your manuscript for publication at this point, I believe that you will be able to address the referees' concerns and I look forward to receiving your revised manuscript. To expedite handling when you resubmit please include a response outlining how you have addressed each of the referees' concerns.

Referee 1:
In this study, the authors have solved the high resolution structures of clathrin TD in complex with various CBM peptides. Surprisingly, in addition to binding to clathrin-box sites, all CBM peptides also showed density at the arrestin box binding region. The new interaction provides a structural basis to explain the redundancy of clathrin recruitment. Moreover, several CBM peptides from amphiphysin or Hepatitis D virus large antigen were found to bind to the Royle box site. This is the first report to confirm the "Royle" binding site at the structural level. They further carry out GST pull down assays in combination with mutagenesis work to evaluate these binding surfaces.

Overall, the manuscript is well-written and the structures support the conclusion that CBM peptide can bind to multiple sites in clathrin NTD. The main limitation of the study is that it does not explore the physiological consequences. Nevertheless, the in vitro and high resolution structural studies more than cross the bar for publication in Traffic.

The authors should consider the following questions. I would encourage but not insist on carrying out further controls experiments to clarify these points.

1) In Figure 2: The unbiased density maps of HDAg-L1, HDAg-L2 in the arrestin box and Royle box are disconnected and do not resolve side chain positions unambiguously. Perhaps this would be clearer at a lower contour level? If the assignments cannot be better supported the authors should be clear about the ambiguity (Figure 4).

The reviewer is correct that the unbiased difference electron density for HDAg-L1 and HDAg-L2 peptides at the Royle box was less extensive than either the density for Amph4T1 at this site or for HDAg-L1 and HDAg-L2 at the clathrin box (Figure 2). However, extensive attempts to model the HDAg-L1 and -L2 peptides in different conformations or orientations failed to yield models with acceptable fit to density and good stereochemistry. We believe that the poor-quality difference density arises from the viral peptides being less well-ordered than Amph4T1 when bound at the Royle box. Comparing the B values of peptides bound at the clathrin, arrestin and Royle boxes with the B values of surrounding residues (Table S1, added to manuscript) confirms that the Amph4T1 peptide bound at the Royle box is better ordered than the HDAg-L peptides, both in absolute terms and when compared to surrounding residues. The same is generally true when comparing the binding of HDAg-L peptides at the Royle box versus the clathrin box. A note to this effect has been added to the Results section.

In terms of illustrating the quality of our peptide models in the vicinity of the Royle box, we fear that reducing the difference density contour level of HDAg-L peptides at the Royle box in Figure 2 may mislead readers as to the strength of our evidence for peptide binding. We have therefore included an additional panel in Figure 4 showing the fit of each refined peptide to a feature-enhanced map [Afonine et al (2015) Acta Cryst. D71, 646-666]. This will allow the reader to get an impression of the model's fit to density that isn't confounded by differences in local map strength (and thus scale of peak heights) between regions of the peptides, with the added benefit that feature-enhanced maps reduce model bias. Lastly, we note that our final refined models and structure factors have been deposited with the PDB and are thus freely available to the community for inspection and re- analysis.

2) In Figure 2: there is clear evidence of CBM peptide in Royle box for Amphiphysin4T1. Since AmphiphysinCBM wasn't predicted to dock in this region, it may indicate that the artificial residues (LERPHRD) may have driven peptide binding to the Royle box. More likely the AmphyiphysinCBM peptide is just not long enough on its own to allow it docking in Royle box and simply needs some flanking residues. This could be tested.

The peptides Amph4T1 and AmphCBM used for crystallisation were the same length, with sequences ETLLDLDFLE and ETLLDLDFDP, respectively. We realise that using the same name for the longer GST-peptide fusion proteins and the peptides used for crystallisation was confusing, for which we apologise. We have modified the manuscript to specifically identify when we're referring to the peptides used for crystallisation (by adding pep to the name of the peptide, e.g. $Amph4T1_{pep}$).

This point notwithstanding, we agree with the reviewer that premature truncation of the peptide used for crystallisation might have prevented binding of a peptide derived from the naturally-occurring amphiphysin sequence at the Royle box. We have therefore crystallised NTD in complex with a longer peptide encompassing the amphiphysin CBM ($AmphCBMlong_{pep}$, sequence ETLLDLDFDPFK). This structure reveals clear binding of the $AmphCBMlong_{pep}$ peptide at the clathrin and arrestin boxes, but not at the Royle box. We thus conclude that is was indeed the non- native residues in $Amph4T1_{pep}$ that facilitated binding of this peptide at the Royle box. We have included details of this new structure solution and refinement, along with figures illustrating $AmphCBMlong_{pep}$ bound at the arrestin and clathrin boxes, but not the Royle box, as supplementary material (Table S2 and Figure S2). Together with the structures of $AP2CBM_{pep}$ and $AmphCBM_{pep}$, the $AmphCBMlong_{pep}$ structure supports our conclusion that the CBM and (as yet undefined) Royle box consensus sequences aren't identical.

3) In the linker of beta2 adaptin (621 IPSQGDLLGD LLNLDLGPPV 640), the sequence contains both a CBM motif and an arrestin box motif ([LI][LI]GxL.). The authors might want to compare which motif favors binding to the arrestin box.

This is an interesting question we hadn't considered – we thank the reviewer for raising it. Given that the peptide motifs overlap (the final leucine of the arrestin box motif being the first of the CBM), a single β2 adaptin molecule could obviously not bind at both sites using both motifs simultaneously.

To address this question we have generated two GST-tagged peptide constructs containing the arrestin box motif of β2 adaptin (AP2arrL and AP2arrS) and compared their ability to capture His- NTD-NEMO to that of the β2 adaptin CBM (AP2CBM). Two GST-arrestin motif constructs were used to avoid issues that might arise from the peptide's carboxylate group immediately following the final leucine of the LLGDL arrestin box motif: AP2arrS ends with the subsequent residue of β2 adaptin (Leu) while AP2arrL ends with the sequence 'AlaSerSer', corresponding to the residues that follow the LLGDL arrestin box motif of arrestin2L [Kang et al (2009)]. To probe relative binding of these peptides to the arrestin box we used the GST fusions as baits to precipitate both wild-type His-NTD-NEMO and a mutant (Q98A+F91K+F9W) where the clathrin and Royle boxes, but not the arrestin box, had been disrupted. We found that AP2CBM and AP2arrS captured wild-type or Q98A+F91K+F9W His-NTD-NEMO more efficiently than did AP2arrL, suggesting that the arrestin consensus motif (LLGDL) alone binds the arrestin box more weakly than does the CBM or an extended arrestin motif (LLGDLL). However, we also noticed that GST-AP2arrL and GST-AP2arrS capture wild-type His-NTD-NEMO much more efficiently than they do the Q98A+F91K+F9W mutant. This strongly suggests that the arrestin consensus motif (LLGDL) is able to bind the clathrin or Royle boxes in addition to binding the arrestin box. This unexpected finding shows that the degeneracy of clathrin binding extends beyond the CBM to the arrestin-box motif. We have included this data as an additional figure in the main text (Figure 6) and a short sections describing this experiment and its implications to the Results and Discussion, respectively.

4) The Amphiphysin sequence also contains two signals: a clathrin box motif and a W box motif (351 LLDLDFDPFK PEVTPAGSAG VTHSPMSQTL PWDLWTTSTD 390). The linker in between is about 20 aa. Miele, et al 2004 showed that the longer peptide in amphiphysin containing these two motifs enhances the binding of clathrin NTD by 6 folds (from 28uM to <5uM) (ref).

The authors may wish to test the longer peptide to check the binding preference.

Our experiments using AmphCBM suggest that the amphiphysin CBM doesn't display a strong preference for binding at the clathrin or arrestin box as we see a significant reduction in binding to NTD only when both sites have been mutated (Figure 5D). We don't expect this result would change in the context of the longer amphiphysin peptide containing both the CBM and W box motifs, since both the arrestin and clathrin boxes would be accessible by a longer amphiphysin peptide bound at the W box site via its W box motif. Specifically, the C-terminal well-ordered residues of the $\text{AmphCBM}_{\text{pep}}$ peptide bound at clathrin and arrestin boxes are 35 Å and 38 Å from the N-terminal residue of the W box motif, respectively. Assuming a span of up to ~3.4 Å per residue in a fully extended conformation, these distances could be easily bridged by the 22–23 residues between the amphiphysin CBM and W box motifs. We therefore respectfully decline to perform this experiment as we fear that our pull down assays would not be sufficiently sensitive to distinguish any slight preference of this longer peptide for the arrestin or clathrin boxes, given that we didn't see a difference in the context of pull down experiments performed using the CBM peptide alone.

Referee: 2
Comments to the Author

Main points.

This detailed structural/biochemistry paper analyses the binding of short, linear 'clathrin box motif' (CBM) peptides to the beta-propeller of the clathrin N-terminal domain (NTD). Perhaps the most significant finding is that NTD binding peptides of beta-2 adaptin, amphiphysin and HDAg-L2 bind to both the clathrin and arrestin box sites (in contrast with previous findings). The authors also defined a fourth peptide binding site on the NTD which they called the 'Royle box'.

This is a clear, straightforward structural paper and I have little to add other than some minor suggestions.

First, it would be helpful to have a broader context (perhaps in the discussion) to frame the detailed information presented. Presumably these different proteins can't all bind the NTD at the same time. How do the authors envisage that the different cellular binding partners 'share time' on the NTD? Is it an ordered, sequential series of binding interactions or some kind of competitive free-for-all?

We thank the reviewer for raising this point. Both the degeneracy of clathrin binding motifs and the kinetics of the association between NTD and such motifs lead us to conclude that, in clathrin-coated pits, there must be rapid turnover of interactions between clathrin and adaptors. This supports the
'free-for-all' model suggested by the reviewer. We have added a paragraph to the Discussion outlining our thoughts regards the dynamic nature of interactions between NTD and adaptor proteins.

Second, how confident are the authors in the 'Royle box' as a 'consensus' binding site given that similar peptides bind in different orientations and that binding does not involve the consensus CBM motif? This is especially true since given the high concentrations of peptides used in the study (~3mM(+)).

Given that all three peptides cover the same patch on the surface of NTD (Figures 4A and 4C), including a hydrophobic cleft, and that the region they cover is highly conserved (Figure 4B), we are quite confident that we have identified a conserved surface on clathrin NTD pre-disposed to mediating protein:protein interactions and capable of binding short peptide motifs. The correspondence between NTD peptide-binding residues our structures and residues that, upon mutation, alter transferrin trafficking in the functional experiments of Willox and Royle (2012) leads us to conclude that we have identified peptide binding at the 'Royle box'. Of course, without knowing the Royle box binding consensus sequence we can't be 100% certain that the binding mode we observe is representative of how a bona fide Royle box motif would bind. However, we hope that presenting these results may stimulate further investigation as to the nature of the Royle-box consensus motif.

With regards to the involvement of the CBM motif in binding at the Royle box, we can be quite confident in asserting that the (unknown) Royle box sequence is distinct from the CBM consensus sequence. As the reviewer states, we used high concentrations of peptide (>3 mM) in all our structural studies. However, for three of the NTD complexes ($AmphCBM_{pep}$, $AP2CBM_{pep}$ and $AmphCBMlong_{pep}$) we see no evidence for peptides bound at the Royle box. Were the CBM and Royle motifs overlapping we would have expected all CBM-containing peptides to also bind at the Royle box. We have added text clarifying this point to the Discussion.

Abstract – Sentence starting 'Surprisingly, with each peptide …'. The sentence doesn't scan, presumably ',' should be replaced by 'and' to read: 'Surprisingly, with each peptide we observe simultaneous peptide binding at multiple sites on the NTD and viral peptides binding to the same sites as cellular peptides.

We thank the reviewer for spotting this flaw and have corrected the text as suggested.

I think 'Fig 5E' is cited before 'Fig 5D'.

We had accidentally included a reference to Figure 5E, which doesn't exist. We've corrected the reference to Figure 5D and thank the reviewer for pointing out this error.
